# Activating Janus charge distribution on the P-doped Ni_3_S_2_/Co_9_S_8_ interface for enhancing charge-matched urea adsorption: boosting high current hydrogen production *via* coupled urine degradation†

**DOI:** 10.1039/d5sc01106j

**Published:** 2025-06-19

**Authors:** Yan Sun, Xiannan Zhang, Hairui Guo, Wenjiang Li, Huiling Liu, Cheng Wang

**Affiliations:** a Tianjin Key Laboratory of Advanced Functional Porous Materials, Institute for New Energy Materials & Low-Carbon Technologies, School of Materials Science and Engineering, Tianjin University of Technology Tianjin 300384 P. R. China cwang@tjut.edu.cn hlliu_tjut2016@163.com; b College of Materials Engineering, Shanxi College of Technology Shuozhou 036000 P. R. China; c Key Laboratory of Display Materials & Photoelectric Devices, School of Materials Science and Engineering, Tianjin University of Technology Tianjin 300384 P. R. China

## Abstract

The urea oxidation reaction (UOR) is emerging as a thermodynamically favorable alternative to the oxygen evolution reaction, offering significant potential for energy-efficient H_2_ production and simultaneous treatment of urea-rich wastewater. However, the 6 e^−^ transfer process of the UOR results in sluggish kinetics, necessitating the development of highly efficient electrocatalysts. Herein, a Janus charge distribution surface is constructed by incorporating phosphorus (P) into the Ni_3_S_2_/Co_9_S_8_ heterojunction to enhance the UOR performance and accelerate urea-assisted H_2_ production. The P incorporation facilitates electron transfer from Ni_3_S_2_ to Co_9_S_8_, creating a local electrophilic/nucleophilic interface that enhances the adsorption of urea molecules with the electron-withdrawing C=O group and electron-donating amino groups. As a result, the modified P-Ni_3_S_2_/Co_9_S_8_ exhibits ultralow potentials of 1.22, 1.30 and 1.39 V (*versus* the reversible hydrogen electrode) to reach 10, 100 and 1000 mA cm^−2^ for the UOR, respectively. Remarkably, when alkaline urine is used as the electrolyte, the P-Ni_3_S_2_/Co_9_S_8_ catalyst, functioning as a bifunctional electrocatalyst in an anion-exchange membrane electrolyzer, can stably deliver a high current density of 1000 mA cm^−2^ for H_2_ production over 180 h. This work highlights the importance of designing electrocatalysts by activating interfacial charge distribution to enhance reactant adsorption and trigger chemical bond cleavage.

## Abstract 2

Incorporating P into Ni_3_S_2_/Co_9_S_8_ creates Janus charge distribution for enhancing urea adsorption and electron transfer, thus boosting the UOR performance. Optimized P-Ni_3_S_2_/Co_9_S_8_ enables efficient urine treatment and H_2_ production *via* HER coupling.

## Graphical abstract

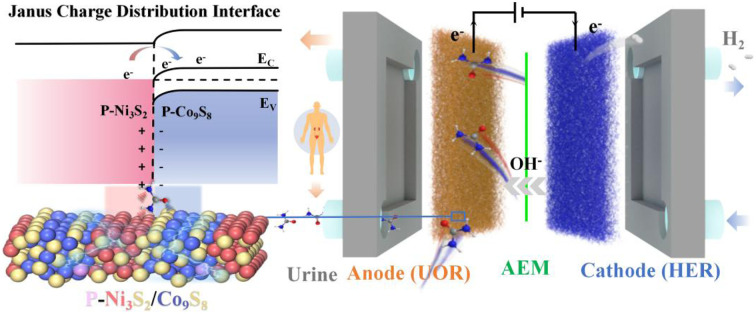

## 1 Introduction

Urea is one of the simplest organic molecules and serves as a crucial component for various life forms. For animals and humans, urea is generated as a nitrogenous end product from the decomposition of proteins and is excreted in urine.^1^ Approximately 265 million tons of urine containing 2–2.5 wt% urea are produced daily, which is more than 500 times the market demand for urea.^2^ After the urine-rich wastewater is released directly into the environment, the untreated urea decomposes naturally into toxic ammonia/nitrate, or nitrogen oxide, causing irreversible environmental damage. Thus, the treatment of urine-rich wastewater has become a major environmental concern.^3,4^ Conventional methods for urea removal, such as adsorption, thermal hydrolysis and chemical oxidation, typically require high energy consumption and are ineffective in treating high loads of urea nitrogen. The electrocatalytic urea oxidation reaction (UOR) offers a promising approach for treating urea/urine wastewater, operating efficiently under ambient conditions with renewable electricity input. Moreover, the UOR has a favorable thermodynamic equilibrium potential of 0.37 V (all potentials are reported *versus* the reversible hydrogen electrode, RHE), compared to the oxygen evolution reaction (OER) of 1.23 V. Theoretically, replacing the OER with the UOR for the coupled hydrogen evolution reaction (HER) can reduce energy consumption for H_2_ production by up to 70%.^5^ In addition to its energy-saving potential, the UOR not only has the potential to save energy but also offers advantages in material accessibility and environmental friendliness. Unlike water electrolysis, which depends on abundant water resources, the UOR utilizes urea from wastewater, converting it into valuable products like hydrogen. This approach not only addresses wastewater management challenges but also promotes resource recycling.^6^ These dual benefits make the UOR a more environmentally friendly and sustainable hydrogen production method compared to traditional water electrolysis.

Despite the thermodynamic advantages, the UOR involves a 6e^−^ transfer step (Co(NH_2_)_2_ + 6OH^−^ → N_2_ + 5H_2_O + CO_2_ + 6e^−^), which results in sluggish reaction kinetics,^5^ making the development of efficient electrocatalysts critical and urgent.^7,8^ In nature, urea decomposition is accelerated by the urease-catalyzed process, where the amine group is bonded to one nickel site of the enzyme and the oxygen atom is attached to another. For the UOR occurring in a heterogeneous electrocatalytic manner, the adsorption behaviors of electrocatalysts to urea are crucial in determining the performance. Accordingly, significant efforts have been made to regulate the adsorption energy or configuration of urea through heteroatom doping,^9–11^ vacancy incorporation^12,13^ and interface engineering.^14,15^ Among these strategies, interface engineering is notable for constructing heterojunctions with adjustable surface states.^16,17^ Strong coupling between different components facilitates electron transfer and redistribution at the interface, forming a Janus charge distribution surface.^18,19^ Given that urea molecules consist of one electron-withdrawing group (C=O) and two electro-donating groups (amino), the electrophilic domain at the interface benefits the –NH_2_ group adsorption, while the nucleophilic domain favors C=O group adsorption. Therefore, constructing heterojunctions and optimizing the charge redistribution at the interface are believed to enhance urea adsorption and improve the UOR performance. Additionally, understanding the adsorption behaviors at the interface for reactants is crucial for guiding the design of active heterojunction electrocatalysts.

Transition metal sulfides, characterized by their unfilled outer d-orbitals and 3d valence electron structure, have emerged as promising catalysts due to their high catalytic activity, low cost, and excellent conductivity.^20^ The recent advances of pentlandites for wastewater treatment further exemplify the versatility and applicability of transition metal sulfides in addressing environmental challenges.^21,22^ These materials exhibit unique advantages in applications such as the organic electro-oxidation reaction,^23^ OER,^24^ and HER.^25^ Recent studies have highlighted their significant potential in bifunctional catalysis, making them ideal alternatives to precious metal catalysts. For example, Mo^4+^-doped NiS has demonstrated remarkable performance in the UOR and HER, attributed to synergistic effects that enhance electron transfer and provide abundant active sites.^9^ Similarly, Fe-modified Ni hydroxysulfide (Fe-NiSOH) nanosheet arrays exhibit rapid self-reconstruction through *in situ* sulfur leaching, promoting the formation of active species.^26^ This design achieves low overpotentials and excellent stability in both water and seawater oxidation. These advancements underscore the potential of transition metal sulfides in developing efficient and durable electrocatalysts for sustainable energy applications.

Herein, we construct a P-modified Ni_3_S_2_/Co_9_S_8_ heterostructure (P-Ni_3_S_2_/Co_9_S_8_) as a bifunctional electrocatalyst for the UOR and HER. The incorporation of P aims to regulate the charge distribution at the interface and further modulate the adsorption behaviors of urea molecules. Experimental results and theoretical calculations indicate that the introduced P promotes electron transfer from Ni_3_S_2_ to Co_9_S_8_ and enhances urea adsorption at the electrophilic (Ni_3_S_2_) and nucleophilic (Co_9_S_8_) interfaces. Benefiting from the modified heterointerface, P-Ni_3_S_2_/Co_9_S_8_ exhibits ultralow potentials of 1.22, 1.30 and 1.39 V at 10, 100 and 1000 mA cm^−2^, respectively, for the UOR. Furthermore, as a bifunctional electrocatalyst, P-Ni_3_S_2_/Co_9_S_8_ requires only 1.70 V to drive 500 mA cm^−2^ in the UOR coupled HER test, which is approximately 500 mV lower than the OER//HER system. Importantly, a flow electrolyzer equipped with the P-Ni_3_S_2_/Co_9_S_8_ electrode and supplied by urine can stably operate at 1000 mA cm^−2^ for over 180 h, further indicating its potential for energy-saving H_2_ production and urine wastewater treatment.

## 2 Results and discussion

### 2.1 Synthesis and characterization

**Fig. 1:**
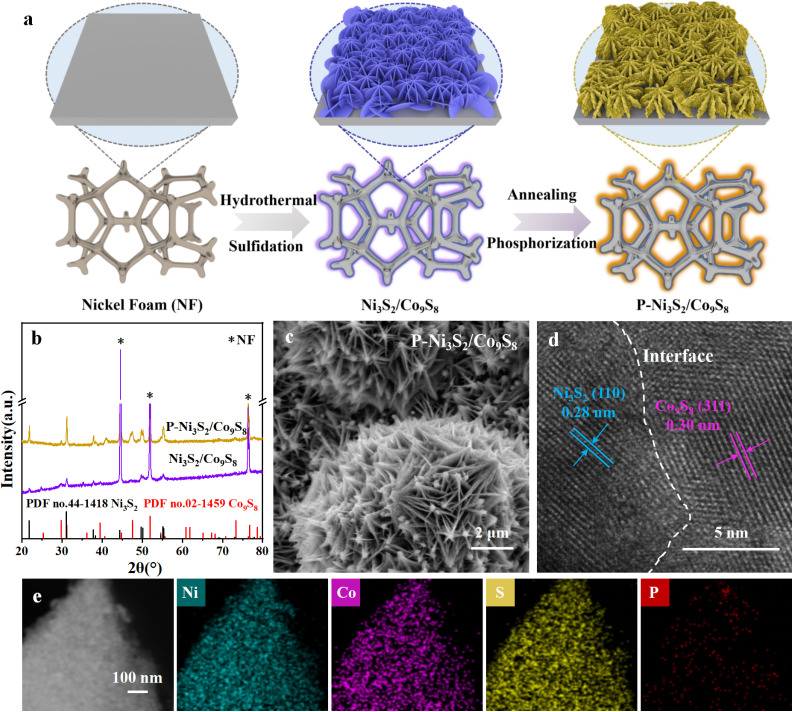
(a) Schematic illustration of the synthetic procedure of P-Ni_3_S_2_/Co_9_S_8_. (b) XRD patterns of Ni_3_S_2_/Co_9_S_8_ and P-Ni_3_S_2_/Co_9_S_8_. (c) SEM image, (d) HRTEM image, and (e) HAADF-STEM image with the corresponding energy-dispersive spectrometry (EDS) mapping images of P-Ni_3_S_2_/Co_9_S_8_.

**Fig. 2:**
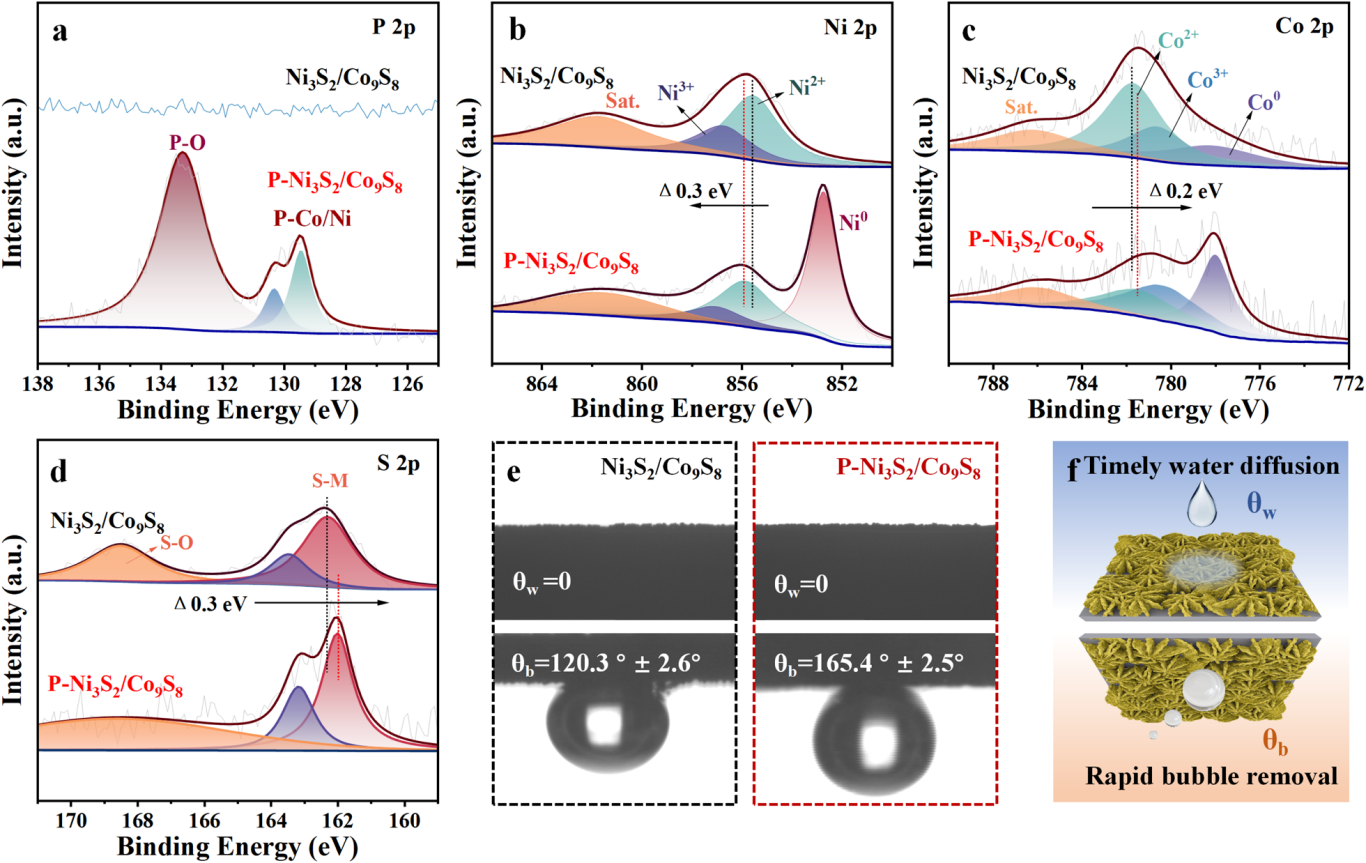
XPS spectra of (a) P 2p, (b) Ni 2p, (c) Co 2p and (d) S 2p for Ni_3_S_2_/Co_9_S_8_ and P-Ni_3_S_2_/Co_9_S_8_. (e) Static water/bubble contact angle images of Ni_3_S_2_/Co_9_S_8_ and P-Ni_3_S_2_/Co_9_S_8_. (f) Schematic illustration of the superhydrophilic and superaerophobic properties of P-Ni_3_S_2_/Co_9_S_8_.

The P-doped Ni_3_S_2_/Co_9_S_8_ heterojunction (P-Ni_3_S_2_/Co_9_S_8_) was synthesized through a two-step process involving a hydrothermal reaction followed by phosphorization treatment, as illustrated in Fig. 1a. Initially, the Ni_3_S_2_/Co_9_S_8_ precursor was prepared on nickel foam (NF) *via* a facile hydrothermal reaction, using NF as the Ni source and substrate and CoCl_2_·6H_2_O and thiourea as the Co and S sources, respectively. Fig. 1b shows the XRD pattern of the Ni_3_S_2_/Co_9_S_8_ precursor. The diffraction peaks at 44.7°, 51.9° and 76.5° are attributed to the NF substrate (PDF no. 04-0850). Additionally, the remaining peaks can be indexed to hexagonal Ni_3_S_2_ (PDF no. 44-1418) and Co_9_S_8_ (PDF no. 02-1459), indicating the coexistence of Ni_3_S_2_ and Co_9_S_8_ phases after hydrothermal synthesis. The Ni_3_S_2_/Co_9_S_8_ precursor exhibits a structure composed of interconnected nanosheets with a smooth surface, grown vertically on the NF substrate, as shown in the scanning electron microscopy (SEM) images (Fig. S1†). After phosphorization treatment, the diffraction peaks of the Ni_3_S_2_ and Co_9_S_8_ phases become more pronounced due to the annealing process at 400 °C for 2 h, while no diffraction peaks from P-related species are detectable (Fig. 1b). Fig. 1c displays that the framework of the nanosheets is basically maintained in P-Ni_3_S_2_/Co_9_S_8_, but the surface becomes rough with the formation of many tiny nanosheets. This hierarchical structure significantly enhances the exposure of the effective surface, thereby promoting contact between reactants and active sites and improving the electrochemical performance.^27^ During the phosphorization process, the doping level of P was controlled by using different amounts of NaH_2_PO_2_ (P content ranging from 5 to 30 mg). Fig. S2, S3,† and 1c present the XRD and SEM results of P_(5,15,20,30)_-Ni_3_S_2_/Co_9_S_8_. As P_(15)_-Ni_3_S_2_/Co_9_S_8_ exhibits the highest electrocatalytic activity, this sample is denoted as P-Ni_3_S_2_/Co_9_S_8_ and used for the following detailed characterization. High-resolution transmission electron microscopy (HRTEM) images of P-Ni_3_S_2_/Co_9_S_8_ (Fig. 1d and S4†) clearly resolve the lattice fringes with spacings of 0.28 and 0.30 nm, corresponding to the (110) plane of Ni_3_S_2_ and (311) plane of Co_9_S_8_, respectively, consistent with the XRD results. Additionally, a clear interface is observed between the adjacent Ni_3_S_2_ and Co_9_S_8_ regions. The high-angle annular dark-field scanning TEM (HAADF-STEM) and the corresponding energy-dispersive spectrometry (EDS) mapping images (Fig. 1e) display the homogeneous distribution of Co, Ni, S, and P elements throughout the nanosheet, with a P-doping amount of approximately 1.01 wt%. These results confirm the successful fabrication of the P-doped Ni_3_S_2_/Co_9_S_8_ heterojunction structure through phosphorization treatment of the Ni_3_S_2_/Co_9_S_8_ precursor. The surface chemical states and valence electron states of P-Ni_3_S_2_/Co_9_S_8_ and Ni_3_S_2_/Co_9_S_8_ were investigated by X-ray photoelectron spectroscopy (XPS). The XPS survey spectra (Fig. S5†) show the presence of P along with Ni, Co and S elements in P-Ni_3_S_2_/Co_9_S_8_, further confirming the incorporation of P through phosphorization treatment. The P 2p spectrum of P-Ni_3_S_2_/Co_9_S_8_ presents two typical species corresponding to P–Co/Ni (130.3 and 129.4 eV) and P–O (133.3 eV)^28^ (Fig. 2a). In the Ni 2p_3/2_ spectrum of P-Ni_3_S_2_/Co_9_S_8_, the peaks at 855.9 and 857.1 eV are assigned to Ni^2+^ and Ni^3+^, while the peak at 852.8 eV is attributed to the metallic Ni^0^ state.^10^ Similarly, the Co 2p_3/2_ spectrum exhibits three peaks at 778.0, 780.3, and 781.5 eV, corresponding to Co^0^, Co^3+^, and Co^2+^, respectively.^29^ The peaks at 162.0 and 163.2 eV observed in the S 2p spectrum of P-Ni_3_S_2_/Co_9_S_8_ can be attributed to S 2p_3/2_ and S 2p_1/2_ of S^2−^ species in Co/Ni sulfide.^30^ Compared with Ni_3_S_2_/Co_9_S_8_, the Ni 2p, Co 2p, and S 2p XPS spectra undergo shifts in binding energy after P-doping (Fig. 2b–d).^31^ Specifically, the Ni 2p peak shifts toward higher binding energy by about 0.3 eV, and the Co 2p peak shifts toward lower binding energy by about 0.2 eV. The binding energy in the S 2p spectrum is reduced by 0.3 eV. These shifts clearly indicate electron transfer from Ni_3_S_2_ to Co_9_S_8_ after P decoration, forming a Janus charge distribution at the P-Ni_3_S_2_/Co_9_S_8_ heterointerface. These results further confirm the successful incorporation of P into Ni_3_S_2_/Co_9_S_8_, which regulates the electronic environment at the heterojunction.

For electrocatalytic reactions proceeding in aqueous electrolyte with gaseous products, the hydrophilic/aerophobic properties of the electrode are essential for practical applications at industrial current densities.^32,33^ As shown in Fig. 2e, both Ni_3_S_2_/Co_9_S_8_ and P-Ni_3_S_2_/Co_9_S_8_ are superhydrophilic, with the static water contact angle (*θ*_w_) close to 0°, enhancing the accessibility of the electrolyte to the catalyst surface during high-current electrochemical reactions. Specifically, the adhesion of gas bubbles to the P-Ni_3_S_2_/Co_9_S_8_ surface is effectively reduced compared to Ni_3_S_2_/Co_9_S_8_. The underwater gas bubble contact angle (*θ*_b_) of P-Ni_3_S_2_/Co_9_S_8_ (165.4 ± 2.5°) is significantly larger than that of Ni_3_S_2_/Co_9_S_8_ (120.3 ± 2.6°) due to the rougher surface of P-Ni_3_S_2_/Co_9_S_8_ after P doping. Moreover, the gas bubbles do not stably adhere to its surface during the test and are dispersed immediately upon contact, showing superaerophobicity.^34^ The superhydrophilic/superaerophobic properties of P-Ni_3_S_2_/Co_9_S_8_ not only ensure intimate contact between the reactants and the electrode surface but also facilitate the quick removal of gaseous products (Fig. 2f), which is critical for stable operation at high current densities.^35^

### 2.2 Electrocatalytic performance for the UOR

**Fig. 3:**
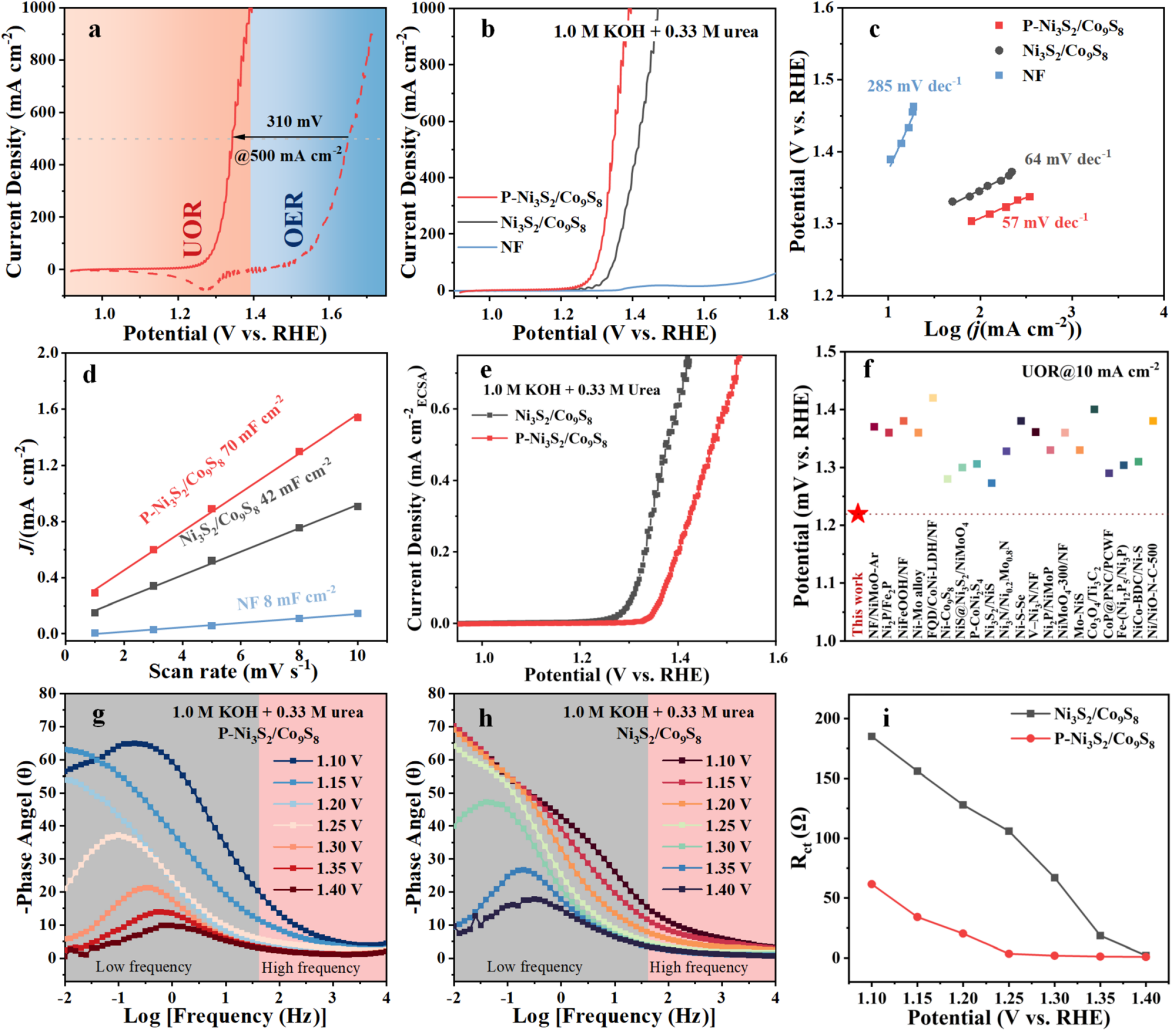
Anodic oxidation performance. (a) LSV curves of P-Ni_3_S_2_/Co_9_S_8_ for the UOR (1.0 M KOH with 0.33 M urea) and the OER (1.0 M KOH). (b) LSV curves, (c) Tafel slopes, and (d) *C*_dl_ values of P-Ni_3_S_2_/Co_9_S_8_, Ni_3_S_2_/Co_9_S_8_, and NF during the UOR. (e) ECSA-normalized LSV curves of P-Ni_3_S_2_/Co_9_S_8_, Ni_3_S_2_/Co_9_S_8_, and NF during the UOR. (f) Comparison of the overpotential for the UOR of our catalyst and other reported Ni/Co-based electrocatalysts. Bode phase plots of (g) P-Ni_3_S_2_/Co_9_S_8_ and (h) Ni_3_S_2_/Co_9_S_8_ at different potentials during the UOR. (i) Fitted *R*_ct_ values of P-Ni_3_S_2_/Co_9_S_8_ and Ni_3_S_2_/Co_9_S_8_.

The electrocatalytic performance of P-Ni_3_S_2_/Co_9_S_8_ for the UOR was investigated in alkaline media with urea as the electrolyte using a standard three-electrode system. All potentials in the linear sweep voltammetry (LSV) polarization measurements were 85% *iR*-corrected and referenced to the reversible hydrogen electrode (RHE), unless otherwise stated. First, the effects of urea and KOH concentrations on the UOR performance were examined. The LSV curves of P-Ni_3_S_2_/Co_9_S_8_ in Fig. S6a† indicate that the UOR performance is relatively insensitive to urea concentration. In contrast, the KOH concentration significantly influences the UOR performance of P-Ni_3_S_2_/Co_9_S_8_, with the polarization current deteriorating as the KOH concentration decreases (Fig. S6b†). The performance remains high between 0.5 and 1.0 M KOH, which is advantageous for treating waste urea resources with varying urea concentrations. In this study, the electrolyte selected for UOR performance measurement was 1.0 M KOH with 0.33 M urea (an average concentration in urine). Subsequently, the influence of P doping level on the UOR performance was investigated, as shown in Fig. S7.† Clearly, the introduction of different amounts of P positively affects the catalyst kinetics of the UOR. Upon comparison, P-Ni_3_S_2_/Co_9_S_8_ with a 15 mg P-doped precursor exhibits the most outstanding UOR performance, characterized by the most significant increase in current and the lowest Tafel slope. Given that the OER is the primary competing reaction for the UOR, the LSV curves of the P-Ni_3_S_2_/Co_9_S_8_ electrocatalyst for the UOR and OER are compared in Fig. 3a. For the OER measurement, the polarization curve was reversely swept to avoid the influence of Ni and Co oxidation.^36^ The current density of the OER starts to increase at 1.45 V and reaches 500 mA cm^−2^ at 1.65 V. In contrast, the LSV curve for the UOR shifts to a much lower potential range. The onset potential is as low as 0.97 V, and P-Ni_3_S_2_/Co_9_S_8_ displays a lower potential of 1.34 V to reach 500 mA cm^−2^. Notably, P-Ni_3_S_2_/Co_9_S_8_ can deliver a high current density of 1000 mA cm^−2^ at a potential of 1.39 V, while the anodic current of the OER is almost negligible at the same potential. These results indicate that the highly active P-Ni_3_S_2_/Co_9_S_8_ can maintain high UOR selectivity at large current densities, effectively mitigating the impact of OER competition. To demonstrate the advantage of P doping in promoting UOR performance, the LSV curves of P-Ni_3_S_2_/Co_9_S_8_ and Ni_3_S_2_/Co_9_S_8_ for UOR are depicted in Fig. 3b. The UOR potentials required for P-Ni_3_S_2_/Co_9_S_8_ to reach 100 and 500 mA cm^−2^ are merely 1.22 and 1.28 V, respectively, which are significantly lower than the 1.33 and 1.37 V required for Ni_3_S_2_/Co_9_S_8_. The NF substrate exhibits negligible activity toward the UOR. In addition, the Tafel slope of P-Ni_3_S_2_/Co_9_S_8_ is determined to be 57 mV dec^−1^, lower than that of the control electrocatalyst (Fig. 3c). The low Tafel slope is also well supported by the observed rapid increase in current density, indicating fast reaction kinetics toward the UOR. The electrochemical surface areas (ECSAs) of P-Ni_3_S_2_/Co_9_S_8_ and Ni_3_S_2_/Co_9_S_8_ were evaluated by calculating their electrochemical double-layer capacitance (*C*_dl_) from the non-faradaic regions of cyclic voltammetry (CV) curves (Fig. S8† and 3d). The *C*_dl_ value of P-Ni_3_S_2_/Co_9_S_8_ is 1.7 and 8.8 times higher than those of Ni_3_S_2_/Co_9_S_8_ and NF, respectively, implying the positive effect of P doping on increasing the ECSA. Moreover, the higher ECSA-normalized current density of P-Ni_3_S_2_/Co_9_S_8_ in Fig. 3e further demonstrates its superior intrinsic activity. The turnover frequency (TOF) was employed to evaluate the intrinsic catalytic activity. The number of active sites was calculated based on the charge capacity obtained by integrating the CV curves (Fig. S9†). Fig. S10† shows that the TOF values of P-Ni_3_S_2_/Co_9_S_8_ consistently exceed those of Ni_3_S_2_/Co_9_S_8_. The high TOF of 8.47 s^−1^, is gained for P-Ni_3_S_2_/Co_9_S_8_ at 1.4 V, which greatly surpasses that of the Ni_3_S_2_/Co_9_S_8_ heterojunction (3.69 s^−1^), further confirming the enhanced UOR intrinsic activity after P doping. The excellent UOR performance of P-Ni_3_S_2_/Co_9_S_8_ surpasses those of the state-of-the-art Co/Ni-based electrocatalysts (Fig. 3f and Table S1†).

To investigate the dynamic evolution of the electrocatalyst interface during the UOR, *in situ* electrochemical impedance spectroscopy (EIS) measurements were performed with the applied potential gradually increased from 1.10 to 1.40 V.^37^Fig. 3g and h show the Bode plots of P-Ni_3_S_2_/Co_9_S_8_ and Ni_3_S_2_/Co_9_S_8_. An apparent phase peak at 1.25 V in the low-frequency region (0.1 Hz) for P-Ni_3_S_2_/Co_9_S_8_ implies the occurrence of a charge transfer process associated with the UOR.^38^ As the applied potential increases, the intensity of the phase angle continuously decreases, and the position of the peak gradually shifts towards higher frequencies. The position of the peak reflects the time domain of the charge transfer process, which is related to the kinetics of the electrochemical reaction.^39^ The phase angle of P-Ni_3_S_2_/Co_9_S_8_ shifts more rapidly towards the high-frequency region compared to Ni_3_S_2_/Co_9_S_8_, indicating the faster catalytic UOR kinetics after P introduction. As displayed in Fig. S11,† the Nyquist plots of P-Ni_3_S_2_/Co_9_S_8_ are steep lines at low applied potentials (<1.25 V), indicating extremely high charge transfer resistances and that the UOR has not yet occurred. The Nyquist plot begins to deviate from the imaginary part after 1.25 V, which is associated with the charge transfer of the UOR process, consistent with the corresponding Bode plots. As the applied potential increases to 1.30 V, significant mutation occurs in the Nyquist plot of P-Ni_3_S_2_/Co_9_S_8_, and a complete small semicircle appears, indicating the start of the UOR.^39^ In contrast, the Nyquist plot of Ni_3_S_2_/Co_9_S_8_ changes at 1.30 V, and a complete semicircle is observed at 1.35 V. Consequently, according to the equivalent circuit (Fig. S12†), Ni_3_S_2_/Co_9_S_8_ shows a later decrease in *R*_ct_ (charge transfer resistance) compared to P-Ni_3_S_2_/Co_9_S_8_ (Fig. 3i), implying faster polarization and oxidation behavior of the UOR on the surface of P-Ni_3_S_2_/Co_9_S_8_.

**Fig. 4:**
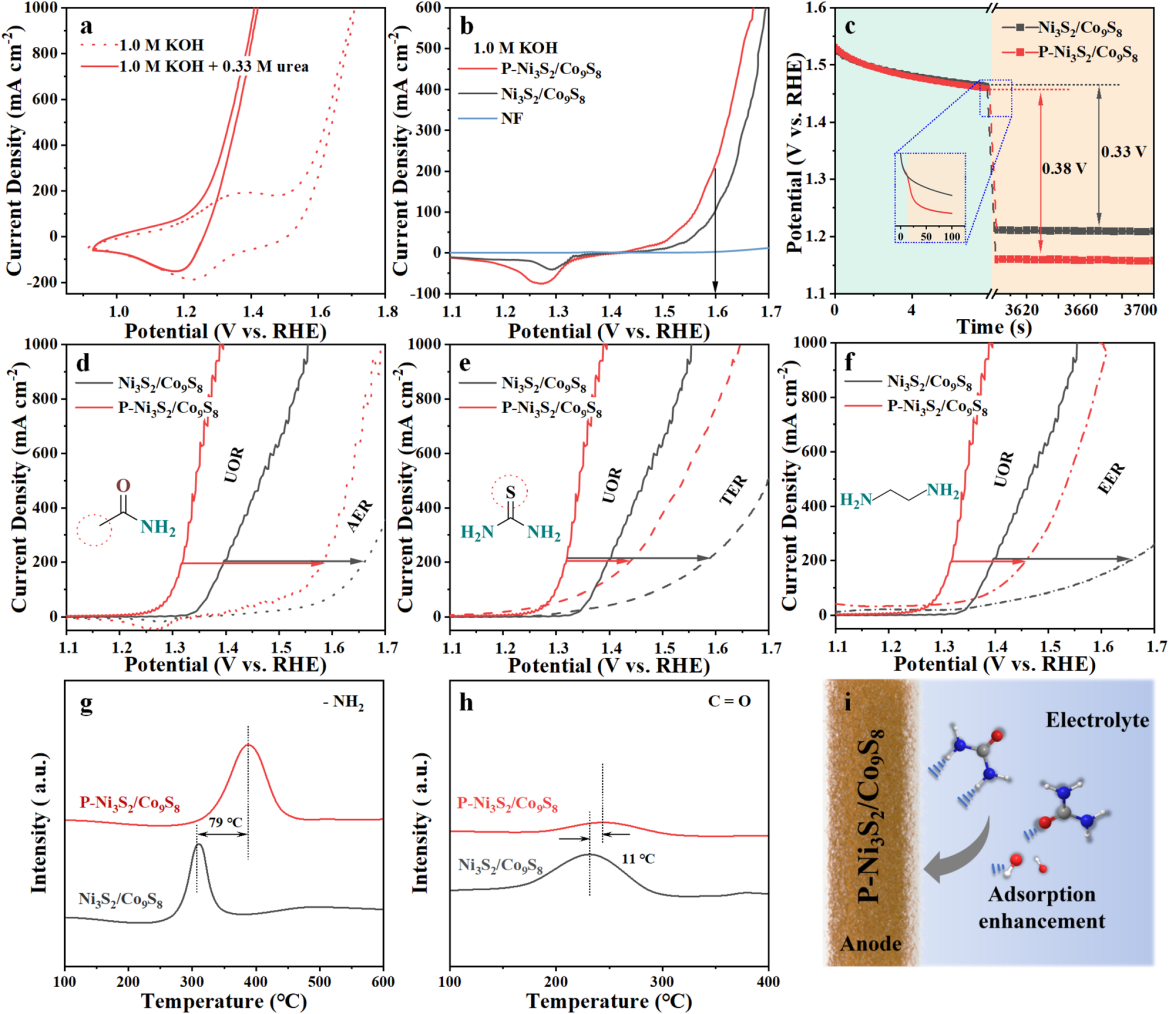
(a) CV curves of P-Ni_3_S_2_/Co_9_S_8_ during the UOR and OER. (b) Backswept LSV curves of P-Ni_3_S_2_/Co_9_S_8_, Ni_3_S_2_/Co_9_S_8_, and NF in 1.0 M KOH. (c) OCP curves of P-Ni_3_S_2_/Co_9_S_8_ and Ni_3_S_2_/Co_9_S_8_ in 1.0 M KOH before and after 0.33 M urea was injected. LSV curves of P-Ni_3_S_2_/Co_9_S_8_ and Ni_3_S_2_/Co_9_S_8_ in 1.0 M KOH with 0.33 M urea or with (d) acetamide, (e) thiourea and (f) ethylenediamine. TPD spectra of P-Ni_3_S_2_/Co_9_S_8_ and Ni_3_S_2_/Co_9_S_8_ in (g) butylamine/He and (h) CO atmospheres. (i) Schematic diagram of the initial reaction in the inner Helmholtz layer.

In light of the UOR electrocatalytic mechanism, the CV curves of P-Ni_3_S_2_/Co_9_S_8_ for the UOR and OER were compared (Fig. 4a). In the CV curve for the OER (1.0 M KOH), a broad oxidation peak is observed at 1.3–1.4 V, corresponding to the oxidation reaction of M^2+^ (M = Co/Ni). Subsequently, a reduction reaction peak of M^3+^ appears during the backward sweep. These observations suggest that P-Ni_3_S_2_/Co_9_S_8_ undergoes pre-oxidation to produce catalytic high-valence species during the OER process, which has been reported for other Co/Ni-based electrocatalysts.^40^ Upon the addition of urea, the oxidation peak disappears, and the current density increases rapidly at the location where the M^2+^ oxidation begins. Following this, a reduction peak appears during the reverse sweep, indicating that the high-valence M^3+^ probably also act as the active species in the UOR process.^41^ Given the similar active sites in the OER and UOR, the adsorption and activation of OH^−^, which is the first step in the OER (OH^−^ + * → OH_ads_ + e^−^),^42^ also plays a crucial role in the UOR in alkaline solutions. Therefore, the adsorption and activation behaviors were detected by evaluating their OER performance. The LSV curves in Fig. 4b show that P-Ni_3_S_2_/Co_9_S_8_ exhibits superior OER performance with a higher polarization current than that of Ni_3_S_2_/Co_9_S_8_. Moreover, in a low OH^−^ concentration environment (Fig. S13†), P-Ni_3_S_2_/Co_9_S_8_ also displays better OER performance. These results imply that the introduction of P could facilitate the effective capture of OH^−^ on the P-Ni_3_S_2_/Co_9_S_8_ surface. Furthermore, changes in the active sites at the electrode–solvent interface were monitored by open-circuit potential (OCP) measurement to evaluate the urea adsorption behavior on the catalyst surface. When 0.33 M urea was injected, the potential equilibrium was disrupted due to the exchange of urea and ions on the catalyst surface, resulting in a significant change in OCP.^43^ In Fig. 4c, the observed change in OCP for P-Ni_3_S_2_/Co_9_S_8_ (0.38 V) is more pronounced compared to Ni_3_S_2_/Co_9_S_8_ (0.33 V), confirming that more urea molecules are adsorbed to the Helmholtz layer of the P-Ni_3_S_2_/Co_9_S_8_ electrode. The faster decrease in OCP for P-Ni_3_S_2_/Co_9_S_8_ upon the addition of urea (inset in Fig. 4c) further provides the evidence of the promoting effect of P on dynamic urea adsorption.^44^

To further investigate the adsorption behavior of Ni_3_S_2_/Co_9_S_8_ and P-Ni_3_S_2_/Co_9_S_8_ towards the two functional groups (–NH_2_ and C=O) of the urea molecule, urea analogues (acetamide, thiourea and ethylenediamine) with similar structural groups were used for comparison. As shown in Fig. 4d–f, the LSV curves for Ni_3_S_2_/Co_9_S_8_ and P-Ni_3_S_2_/Co_9_S_8_ in the acetamide electrocatalytic oxidation reaction (AER), thiourea electrocatalytic oxidation reaction (TER), and ethylenediamine electrocatalytic oxidation reaction (EER) were shifted to higher potentials compared to the UOR, indicating a decrease in performance. In particular, the degradation caused by the –NH_2_ alteration (AER) was much more pronounced than that caused by the C=O alteration (TER). P-Ni_3_S_2_/Co_9_S_8_ showed the least degradation performance in the EER (Fig. 4f), which has two –NH_2_ groups like the urea molecule, but Ni_3_S_2_/Co_9_S_8_ showed significant degradation. As displayed in Fig. S14,† the results of the OCP tests also showed that P-Ni_3_S_2_/Co_9_S_8_ has a significantly lower adsorption strength in the absence of the –NH_2_ group (AER). Compared to Ni_3_S_2_/Co_9_S_8_, P-Ni_3_S_2_/Co_9_S_8_ exhibits higher –NH_2_ adsorption (TER and EER). These results suggest that the improved performance of P-Ni_3_S_2_/Co_9_S_8_ is likely due to its enhanced adsorption strength of –NH_2_ during the oxidation reaction. In parallel, programmed temperature rise desorption (TPD) tests were conducted on P-Ni_3_S_2_/Co_9_S_8_ and Ni_3_S_2_/Co_9_S_8_ to investigate the adsorption behavior towards different groups of urea molecules. In a butylamine/He atmosphere, the desorption temperature for P-Ni_3_S_2_/Co_9_S_8_ is higher than that for Ni_3_S_2_/Co_9_S_8_, demonstrating stronger adsorption of P-Ni_3_S_2_/Co_9_S_8_ for the –NH_2_ group (Fig. 4g). The desorption temperature of P-Ni_3_S_2_/Co_9_S_8_ for urea molecules in the CO-TPD test was similar to that of Ni_3_S_2_/Co_9_S_8_ (Fig. 4h), but the desorption temperature of P-Ni_3_S_2_/Co_9_S_8_ was slightly higher than that of Ni_3_S_2_/Co_9_S_8_. These results suggest that the enhanced urea adsorption should be mainly attributed to the –NH_2_ group, which is consistent with the LSV test results for urea analogue substrates (Fig. 4i).

The surface electronic structures and chemical states of P-Ni_3_S_2_/Co_9_S_8_ were investigated using XPS after the OER and UOR tests (Fig. S15†). After the OER test, the peak intensity of the P/S element is considerably decreased, and the signals of P/S-Co/Ni disappear. Compared to the initial P-Ni_3_S_2_/Co_9_S_8_, the surface concentrations of Ni^0/2+^ and Co^0/2+^ species are significantly lower after the OER test, while the ratios of Ni^3+^ and Co^3+^ increase. This suggests that the Ni^0/2+^ and Co^0/2+^ species were oxidized to Ni^3+^ and Co^3+^, respectively.^45^ Furthermore, the O 1s spectrum reveals that the characteristic peaks corresponding to O-MO_*x*_ species become dominant after the OER test, confirming the reconstruction of the catalyst surface into metal oxyhydroxide species. In contrast, the XPS results after the UOR test are significantly different from those after the OER test. The proportion of P/S–O characteristic peaks increase after the UOR test, although a certain proportion of P/S–Co/Ni remains detectable, implying partial surface oxidation on the catalyst. The peaks corresponding to Ni^0/3+^ species disappear, and only Ni^2+^ species are present. For the Co element, the Co^3+^/Co^2+^ ratio does not change significantly. Similarly, the O 1s spectrum exhibits no significant changes. Based on these results, it is proposed that the proton in the urea molecule may fill the hydrogen defect in the intermediate metal hydroxyl species, thereby avoiding a phase transition to metal oxyhydroxide. This finding is consistent with the previously reported nucleophilic oxidation mechanism mediated by metal redox pairs (M^2+^/M^3+^).^46^

**Fig. 5:**
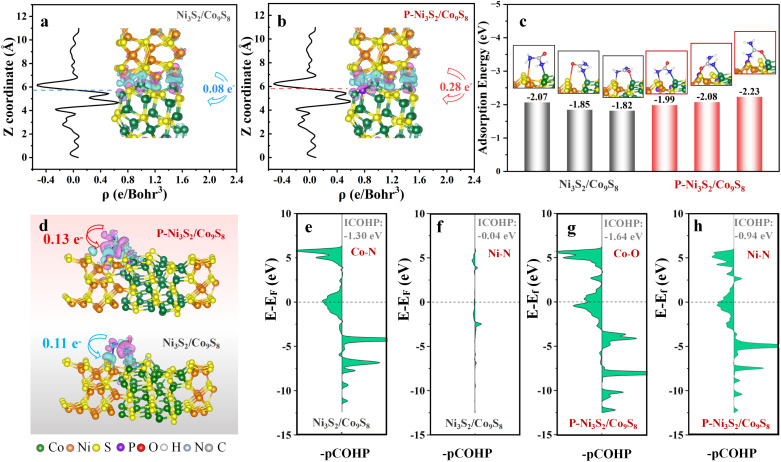
Average charge density difference of (a) Ni_3_S_2_/Co_9_S_8_ and (b) P-Ni_3_S_2_/Co_9_S_8_. (c) Three optimized adsorption structures and adsorption energies for CO(NH_2_)_2_ on Ni_3_S_2_/Co_9_S_8_ and P-Ni_3_S_2_/Co_9_S_8_. (d) The difference charge density and Bader charge analysis for CO(NH_2_)_2_ on Ni_3_S_2_/Co_9_S_8_ and P-Ni_3_S_2_/Co_9_S_8_, where the isosurface value is set to 0.0015 e Å^−3^ and the positive and negative charges are shown in cyan and magenta, respectively. The green, orange, yellow, purple, red, white, blue, and gray balls represent Co, Ni, S, P, O, H, N and C atoms, respectively. Projected crystal orbital Hamiltonian population (pCOHP) analysis between CO(NH_2_)_2_ and (e) and (f) Ni_3_S_2_/Co_9_S_8_ and (g) and (h) P-Ni_3_S_2_/Co_9_S_8_.

Density functional theory (DFT) calculations were performed to elucidate the effectiveness of P decoration in enhancing the UOR performance of the P-Ni_3_S_2_/Co_9_S_8_ heterostructure. Based on experimental results and theoretical calculations, the optimized model for P-Ni_3_S_2_/Co_9_S_8_, where surface S atoms are partially replaced by P atoms, was employed for the following DFT study (Fig. S16†).^47,48^ The Ni_3_S_2_/Co_9_S_8_ model is similar to that of P-Ni_3_S_2_/Co_9_S_8_, except for the absence of P atom substitution. The effect of P incorporation on the charge distribution at the Ni_3_S_2_/Co_9_S_8_ heterointerface was first investigated *via* analyzing the charge density difference. Fig. 5a and b indicate that electrons transfer from Ni_3_S_2_ to Co_9_S_8_. Upon P incorporation, the charge distribution significantly increases from 0.08 e^−^ (Ni_3_S_2_/Co_9_S_8_) to 0.28 e^−^ (P-Ni_3_S_2_/Co_9_S_8_), forming a Janus charge distribution interface with electrophilic P-Ni_3_S_2_ and nucleophilic P-Co_9_S_8_. This Janus interface facilitates the selective adsorption of urea's electron-withdrawing group (C=O) and electron-donating group (amino). For heterogeneous electrocatalysis, the adsorption behaviors of reactants on electrocatalysts typically play a crucial role in affecting the corresponding reaction processes. To determine the adsorption behaviors of urea, all possible NN-terminal and NO-terminal adsorption configurations at the interfaces of Ni_3_S_2_/Co_9_S_8_ and P-Ni_3_S_2_/Co_9_S_8_ were considered (Fig. S17†). As compared in Fig. 5c, the stable adsorptions for each sample were screened according to the calculated adsorption energies. Accordingly, urea tends to adsorb as a NN-terminal configuration with an adsorption energy of −2.07 eV at the interface of Ni_3_S_2_/Co_9_S_8_. For P-Ni_3_S_2_/Co_9_S_8_, a NO-terminal configuration (N adsorbed on the Ni site and O adsorbed on the Co site) with a more negative adsorption energy of −2.23 eV is favorable, confirming enhanced urea adsorption upon P incorporation. Additionally, the local charge density difference for Ni_3_S_2_/Co_9_S_8_ and P-Ni_3_S_2_/Co_9_S_8_ adsorbed urea was calculated, and the results are shown in Fig. 5d. Upon Bader charge analysis, the P-decorated heterojunction displays a larger charge density difference of 0.13 e^−^ compared to 0.11 e^−^ for Ni_3_S_2_/Co_9_S_8_. Thus, the incorporation of P not only increases the charge density difference but also promotes electron transfer from the adsorbed urea molecule to the P-Ni_3_S_2_/Co_9_S_8_ interface, which is crucial for enhancing urea adsorption. Thus, the incorporation of P favors electron transfer from the adsorbed urea molecule to P-Ni_3_S_2_/Co_9_S_8_, thereby enhancing urea adsorption. To further quantitatively investigate the bonding strengths between active sites and the adsorbed urea, crystal orbital Hamiltonian population (COHP) analysis was carried out.^49^Fig. 5e–h compare the integrated COHP (ICOHP) calculated by integrating COHP over all levels up to the Fermi level. The ICOHP for P-Ni_3_S_2_/Co_9_S_8_ (−1.64 and −0.94 eV) is more negative compared to Ni_3_S_2_/Co_9_S_8_ (−1.30 and −0.04 eV), further confirming a stronger coupling between the transition metal (Ni/Co) and urea (N/O) orbitals upon P incorporation. This stronger orbital coupling, induced by P incorporation, directly contributes to the enhanced adsorption and activation of urea molecules on the heterojunction surface. All these results indicate that the incorporation of P leads to modification in the electronic structure of the heterojunction, thereby enhancing adsorption and promoting UOR activity.

### 2.3 Electrocatalytic performance for the HER

**Fig. 6:**
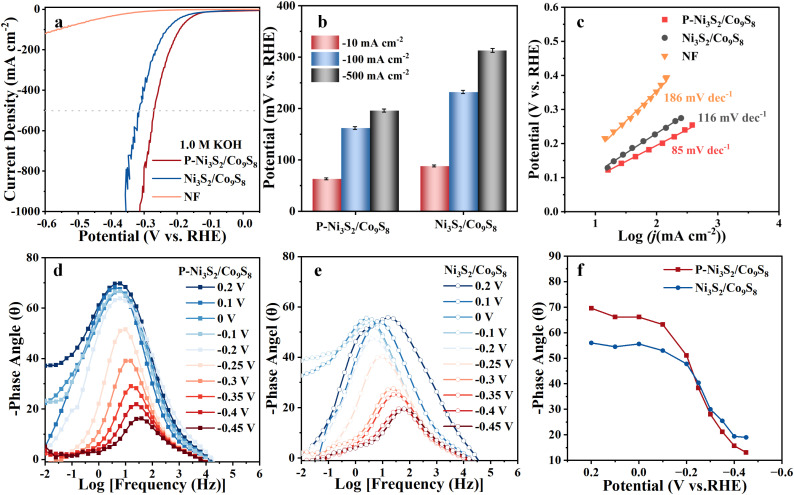
Electrocatalytic HER performance. (a) LSV curves of P-Ni_3_S_2_/Co_9_S_8_, Ni_3_S_2_/Co_9_S_8_, and NF in 1.0 M KOH. (b) Comparison of potentials at different current densities between P-Ni_3_S_2_/Co_9_S_8_ and Ni_3_S_2_/Co_9_S_8_. (c) Tafel slopes of P-Ni_3_S_2_/Co_9_S_8_, Ni_3_S_2_/Co_9_S_8_, and NF for the HER. *In situ* EIS characterization. Bode phase plots of (d) P-Ni_3_S_2_/Co_9_S_8_ and (e) Ni_3_S_2_/Co_9_S_8_ at different potentials during the HER. (f) Corresponding response of the phase angle to the applied potential.

In addition to its remarkable UOR activity, the electrocatalytic HER performance of the P-Ni_3_S_2_/Co_9_S_8_ was also evaluated in an alkaline electrolyte (1.0 M KOH) using a three-electrode system. The LSV curves in Fig. 6a show that the potential of P-Ni_3_S_2_/Co_9_S_8_ is significantly lower than that of Ni_3_S_2_/Co_9_S_8_ in the current density range of 0–1000 mA cm^−2^. Specifically, to achieve current densities of 10, 100, and 500 mA cm^−2^, P-Ni_3_S_2_/Co_9_S_8_ requires overpotentials of 63, 162, and 192 mV, respectively, which are much lower than those of Ni_3_S_2_/Co_9_S_8_ (88, 232, and 313 mV, Fig. 6b). Meanwhile, P-Ni_3_S_2_/Co_9_S_8_ shows a significantly lower Tafel slope of 85 mV dec^−1^ compared to 116 mV dec^−1^ for Ni_3_S_2_/Co_9_S_8_ (Fig. 6c), indicating faster HER kinetics on the surface of P-Ni_3_S_2_/Co_9_S_8_. Meanwhile, a high TOF value of 7.08 s^−1^ can be achieved at −0.2 V for P-Ni_3_S_2_/Co_9_S_8_ (Fig. S18†), which is 4.9 times higher than that of Ni_3_S_2_/Co_9_S_8_, demonstrating its exceptionally high intrinsic catalytic activity. The HER performance of P-Ni_3_S_2_/Co_9_S_8_ exhibited a satisfactory level. Compared with reported Ni-based catalysts, although it did not reach the optimum, it was on a par with the upper-middle range (Table S1†). Furthermore, *in situ* EIS was performed to further probe the alkaline HER kinetics. As shown in Fig. 6d and e, the Bode plots for P-Ni_3_S_2_/Co_9_S_8_ and Ni_3_S_2_/Co_9_S_8_ show similar trends in phase angles, while P-Ni_3_S_2_/Co_9_S_8_ exhibits a greater downward trend in the phase angle response due to more efficient electron transfer (Fig. 6f).^50^ The Nyquist plots of P-Ni_3_S_2_/Co_9_S_8_ and Ni_3_S_2_/Co_9_S_8_ at potentials of 0.2–0.45 V show a gradual shift from a steep line to a semicircule (−0.2 V) as the applied potential increases (Fig. S19†). Subsequently, the semicircle diameter becomes progressively smaller as the potential increases, suggesting that the reaction rate increases. P-Ni_3_S_2_/Co_9_S_8_ consistently has a smaller semicircular diameter than Ni_3_S_2_/Co_9_S_8_ at the same potential, further confirming the faster electron transfer at the P-Ni_3_S_2_/Co_9_S_8_ interface. Consequently, the introduction of P in the heterojunction structure enhances electron transfer and accelerates the kinetics of the HER. The HER activity of P-Ni_3_S_2_/Co_9_S_8_ was also evaluated in a 1.0 M KOH solution containing urea to verify the feasibility of UOR as an alternative to the OER for coupling with the HER. As shown in Fig. S20,† the nearly overlapping LSV curves indicate that urea has a negligible effect on the HER performance of P-Ni_3_S_2_/Co_9_S_8_, ensuring its potential application in the energy-saving UOR coupled H_2_ production.

### 2.4 Electrocatalytic activity toward overall flow-mode urea electrolysis

**Fig. 7:**
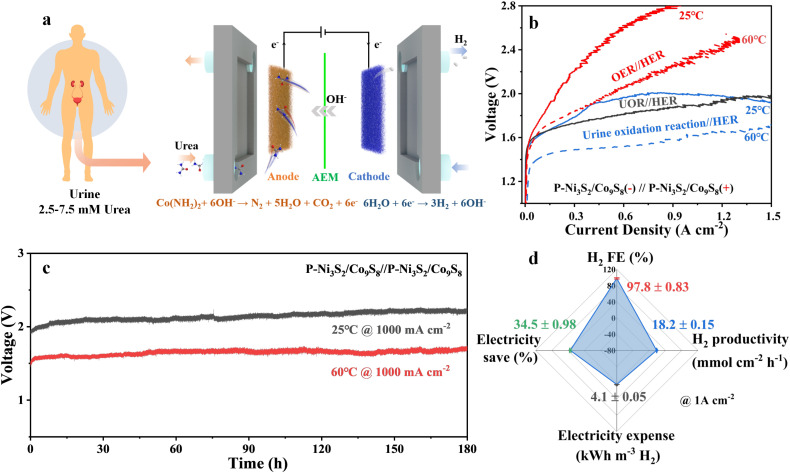
Urea-assisted electrolysis performance in an AEM electrolyzer. (a) Schematic illustration of the urea electrolyzer using P-Ni_3_S_2_/Co_9_S_8_ as both the anode and cathode. (b) Polarization curves of P-Ni_3_S_2_/Co_9_S_8_(−)//P-Ni_3_S_2_/Co_9_S_8_(+). (c) Durability cell voltage–time plots for P-Ni_3_S_2_/Co_9_S_8_(−)//P-Ni_3_S_2_/Co_9_S_8_(+) at a constant current density of 1000 mA cm^−2^ (the inset shows the photographs of the AEM electrolyzer). (d) The comprehensive performance of P-Ni_3_S_2_/Co_9_S_8_.

Considering the outstanding bifunctional electrocatalytic activities of P-Ni_3_S_2_/Co_9_S_8_ towards the UOR and HER, we constructed a paired UOR//HER two-electrode cell, employing P-Ni_3_S_2_/Co_9_S_8_ as both the anode and cathode (Fig. S21†). As depicted in Fig. S21b,† the UOR//HER system in 1.0 M KOH containing 0.33 M urea exhibits a significantly lower onset potential and a more rapid current increase compared to the OER//HER system in 1.0 M KOH. Specifically, to achieve a high current density of 500 mA cm^−2^, the UOR//HER system requires a potential of only 1.70 V, approximately 500 mV lower than that of the OER//HER system. These results underscore the potential of the bifunctional P-Ni_3_S_2_/Co_9_S_8_ for urea-assisted electrolysis as an energy-saving H_2_ production pathway, potentially replacing conventional water electrolysis. Inspiringly, to further evaluate the practicality of P-Ni_3_S_2_/Co_9_S_8_ for urea-assisted H_2_ production, an anion exchange membrane (AEM) flow electrolyzer was assembled, effectively enhancing mass transfer and reducing concentration polarization (Fig. 7a and S22†). When using artificial urine (with pH adjusted to 14) as the electrolyte, the LSV curve may fluctuate due to the presence of impurities such as Cl^−^, Mg^2+^, and Ca^2+^. The flow-mode UOR//HER device operates efficiently, demonstrating a significantly reduced cell voltage compared to the OER//HER (Fig. 7b). At 25 °C and a high current density of 1000 mA cm^−2^, the flow-mode UOR//HER device achieves a 34.5 ± 0.98% reduction in electricity consumption relative to the OER//HER system, demonstrating the effectiveness of the UOR//HER system in minimizing energy expenditure.^51^ The stability of an electrocatalyst under high current density is a crucial metric for evaluating in practical applications. As illustrated in Fig. 7c, the P-Ni_3_S_2_/Co_9_S_8_ catalyst exhibits excellent stability across different temperatures. At 25 °C, it maintains 92.1% of its initial voltage after 180 hours of continuous operation at 1000 mA cm^−2^. Initially, a slight voltage increase is observed, which can be attributed to pre-oxidation effects.^52^ Subsequently, the voltage increases gradually, likely due to the gradual depletion of urea concentration and minor influences from urine components on the catalyst. However, these factors have a minimal impact on the overall performance. Notably, the degradation rate of 0.044% corresponding to the 92.1% voltage retention is significantly better than those reported in several literature references (Table S2†), where higher degradation rates are typically observed under similar conditions. Furthermore, the XRD pattern, SEM image, and EDS spectrum of P-Ni_3_S_2_/Co_9_S_8_ following the durability test exhibit negligible variation in morphology, phase, and composition (Fig. S23 and S24†). The potential leaching of Ni and Co from the P-Ni_3_S_2_/Co_9_S_8_ catalyst into the electrolyte was detected with ICP-AES. Following a 180 h durability test, the amount of dissolved Ni and Co was found to be less than 1.0 μg cm^−2^ (corresponding to the geometric area of the electrode, Table S3†). This value is significantly lower than the Ni and Co amounts in the pristine P-Ni_3_S_2_/Co_9_S_8_, thereby effectively ruling out the possibility of substantial dissolution of these metals from the catalyst. These findings reliably demonstrate that P-Ni_3_S_2_/Co_9_S_8_ possesses outstanding stability for the UOR. The faradaic efficiency (FE) of H_2_ production was monitored during the stability test by periodically comparing the amount of theoretical and measured H_2_ (Fig. S25†). The FE for H_2_ is maintained at a high level (97.8 ± 0.83%), with a productivity of 18.2 ± 0.15 mmol cm^−2^ h^−1^, accompanied by a low energy consumption of 4.1 ± 0.05 kWh m^−3^ H_2_ (Fig. 7d). More impressively, when the temperature is increased to 60 °C, the catalyst not only exhibits remarkable enhancement in performance but also maintains outstanding stability, achieving a voltage retention of 93.5%. Gas chromatography (GC) analysis was conducted on the anode gaseous products at different time intervals. The results reveal the generation of N_2_ and O_2_ during the UOR process (Fig. S26 and Table S4†). Here, O_2_ is produced through the competitive OER. Notably, N_2_ is identified as the predominant product, exhibiting a high FE of 71.29 ± 1.67%, while the FE of O_2_ is only 0.51 ± 0.02%. This indicates that the majority of electrons are utilized for catalyzing urea oxidation rather than the OER. Analysis of the anolyte by ion chromatography (IC) reveals that the primary liquid product is NO_2_^−^ (Fig. S27†), with a FE of 37.52 ± 1.90%. Therefore, taking all factors into account, after the long-term stability test, the main products from the UOR were N_2_ and NO_2_^−^.^53^ Overall, given its remarkable activity and stability, the P-Ni_3_S_2_/Co_9_S_8_ electrocatalyst holds great promise for industrial application in energy-saving H_2_ production *via* urea-assisted electrolysis.

## 3 Conclusions

In summary, a P-modified Ni_3_S_2_/Co_9_S_8_ heterostructure with a Janus charge distribution interface is constructed as an efficient electrocatalyst for urea-assisted H_2_ production. The incorporation of P effectively enhances the electron transfer from Ni_3_S_2_ to Co_9_S_8_ at the interface. The resultant electrophilic P-Ni_3_S_2_ and nucleophilic P-Co_9_S_8_ domains facilitate the adsorption of electron-donating amino and electron-withdrawing carbonyl groups of urea, thereby modulating the adsorption energy and interaction. Consequently, the prepared P-Ni_3_S_2_/Co_9_S_8_ exhibits enhanced UOR performance with low potentials of 1.22, 1.30, and 1.39 V (*vs.* RHE) at current densities of 10, 100, and 1000 mA cm^−2^, respectively. As a bifunctional electrocatalyst, P-Ni_3_S_2_/Co_9_S_8_ also displays excellent HER activity, requiring a minimal potential of 65 mV at 10 mA cm^−2^. Accordingly, an AEM flow electrolyzer equipped with this bifunctional P-Ni_3_S_2_/Co_9_S_8_ and supplied with alkaline urine enables stable H_2_ production at 1000 mA cm^−2^ for 180 h. Compared to conventional water splitting, the UOR//HER system achieves a satisfactory H_2_ productivity of 18.2 mmol cm^−2^ h^−1^ with 34.5% electricity saving. This work presents a powerful strategy for activating the Janus charge distribution at heterojunction interfaces, thereby optimizing the adsorption behaviors of reactants and improving the performance.

## Author contributions

C. W. (funding acquisition: lead; resources: lead; writing – review & editing: equal); H. L. (funding acquisition: equal; resources: equal; validation: equal; writing – review & editing: equal); Y. S. (conceptualization: lead; data curation: lead; investigation: lead; methodology: lead; project administration: supporting; validation: lead; visualization: lead; writing – original draft: lead); X. Z. (data curation: supporting; investigation: supporting; validation: supporting); H. G. (data curation: supporting; formal analysis: supporting; validation: supporting); W. L. (funding acquisition: supporting; validation: supporting; writing – review & editing: supporting).

## Conflicts of interest

There are no conflicts to declare.

## Supplementary Material

SC-OLF-D5SC01106J-s001

## Data Availability

All data can be found in the main article or the ESI.†
