# Supplementary material for: Activating Janus charge distribution on the P-doped Ni3S2/Co9S8 interface for enhancing charge-matched urea adsorption: boosting high current hydrogen production via coupled urine degradation
Source: Chem Sci. 2025 Jun 19;16(29):13401–12. doi: 10.1039/d5sc01106j (PMC12189000; doi:10.1039/d5sc01106j)
Supplement: SC-016-D5SC01106J-s001 [file SC-016-D5SC01106J-s001.pdf]

Supplementary Information (SI) for Chemical Science.
This journal is © The Royal Society of Chemistry 2025

Activating Janus Charge Distribution on P-Doped Ni_3S_2/Co_9S_8 Interface for Enhancing Charge-Matched Urea Adsorption: Boosting High Current Hydrogen Production via Coupled Urine Degradation

Yan Sun,[ab] *Xiannan Zhang,*[a] *Hairui Guo,*[a] *Wenjiang Li,*[c] *Huiling Liu**[a] *and Cheng Wang**[a]

Y. Sun, H. Guo, X. Zhang, H. Liu and C. Wang

[a] Tianjin Key Laboratory of Advanced Functional Porous Materials, Institute for New Energy Materials & Low-Carbon Technologies, School of Materials Science and Engineering, Tianjin University of Technology, Tianjin 300384, P.R. China

Y. Sun

[b] Shanxi College of Technology, College of Materials Engineering, Shuozhou 036000, Shanxi Province, P.R. China

W. Li

[c] Key Laboratory of Display Materials& Photoelectric Devices, School of Materials Science and Engineering, Tianjin University of Technology, Tianjin 300384, P.R. China

1. Experiment Section

1.1. Chemical and Materials

Potassium hydroxide (KOH), cobaltous chloride ($CoCl_2 \cdot 6H_2O$), ethanol, thiourea, and urea were obtained from Sigma-Aldrich Corporation. Sodium monophosphate (NaH_2PO_2) and hydrochloric acid (HCl) were sourced from National Pharmaceutical Group Co., Ltd. Nickel foam (thickness 1.6 mm) was purchased from Suzhou Shuertai Industrial Technology Co., Ltd., China. All chemicals were of analytical grade and were used without further purification. Artificial urine (pH 6.5) was provided by Chang Feng Technology Co..

1.2. Synthesis of Ni_3S_2/Co_9S_8

Ni_3S_2/Co_9S_8 heterojunctions were synthesized via a facile one-step hydrothermal process. Typically, a piece of NF (2.0 × 3.0 cm^2) was ultrasonicated for 10 min in an aqueous solution of 3.0 M HCl, ethanol and ultrapure water, respectively. For the synthesis of the Ni_3S_2/Co_9S_8 sample, $CoCl_2 \cdot 6H_2O$ (2 mmol) and thiourea (4 mmol) were dissolved in 30 mL of ultrapure water. The resulting solution was transferred to a stainless-steel Teflon-liner autoclave (50 mL). The NF was vertically immersed into the autoclave reactor.

The hydrothermal reaction was carried out at 160°C for 12 h. The prepared Ni_3S_2/Co_9S_8 was collected, thoroughly rinsed with ultrapure water, and dried in a vacuum oven at 60°C for 12 h.

1.3. Synthesis of P-doped Ni_3S_2/Co_9S_8

The synthesized Ni_3S_2/Co_9S_8 was placed in the downstream section of tube furnace, while 100 mg NaH_2PO_2 was put upstream side. The sample was heated to 400℃ at a rate of 10℃/min for 2 h under Ar gas flow of 80 mL min^{-1} to obtain P-Ni_3S_2/Co_9S_8. To further investigate the effect of phosphorus content, comparison samples $P_{(5)}$-Ni_3S_2/Co_9S_8, $P_{(20)}$-Ni_3S_2/Co_9S_8 and $P_{(30)}$-Ni_3S_2/Co_9S_8 were prepared using a similar method to but with varying amounts of phosphorus (5, 20 and 30 mg, respectively).

2. Materials characterization

The structures and morphologies of the prepared samples were characterized by X-ray diffraction (XRD, SmartLab 9KW, Rigaku, Japan), scanning electron microscope (SEM, Verios 460L, USA), and Transmission electron microscopy (TEM, TECNAL G2 Spirit TWIN) equipped with a LaB6 emission gun. The surface chemical composition and valence states of the materials were determined through X-ray photoelectron spectroscopy (XPS, ESCALAB250Xi, THERMO SCIENTIFIC, UK). The water droplet and underwater gas-bubble contact angle of the samples were recorded using KRüSS-DSA100 (Germany) contact angle meter. Temperature programmed desorption (TPD) of CO and butylamine were performed on a Micromeritics Autochem II 2920 chemisorption analyzer. For each run, the sample was heated to 423 K for 0.5 h at a rate of 10 K min^{-1} in a He flow to remove adsorbed impurities. The sample was then cooled down to 313 K for the adsorption of CO gas (99.999%). After purging with He (99.999%) for 1 h to remove physically adsorbed CO, the TPD data were collected from 313 K to 873 K with a ramp of 10 K min^{-1} in the TCD detector. The TPD measurement of butylamine followed a similar procedure to that of CO, except that butylamine was carried by He (99.999%) gas into the catalysts until adsorption saturation and subsequently desorbed up to 473 K in the He

atmosphere. The Ni and Co contents were quantified by inductivity coupled plasma-atomic emission spectrometry (ICP-AES, Thermo Scientific).

3. Electrochemical Measurements

Electrochemical measurements for the UOR, OER, HER, Acetamide electrooxidation reaction (AER), Thiourea electrooxidation reaction (TER) and Ethylenediamine electrooxidation reaction (EER) were conducted on a Metrohm Autolab PGSTAT302N electrochemical workstation in an H-type cell, separated by a Nafion 117 proton exchange membrane. The electrochemical studies utilized a conventional three-electrode configuration, with the as-synthesized catalyst on Ni foam (1.0 × 1.0 cm^2), Hg/HgO, and a graphite rod were used as the working electrode, reference, and counter electrode, respectively. Linear sweep voltammetry (LSV) curves were performed at a scan rate of 5 mV/s until the results stabilized. All polarization curves were corrected using 85% iR compensation and converted against RHE through the following equation:

$$E_{vs.\ RHE} = E_{vs.\ Hg/HgO} + 0.098\ \text{V} + 0.0592\ \text{pH} \qquad \text{(S1)}$$

Where $E_{vs.\ RHE}$ and $E_{vs.\ Hg/HgO}$ are the potentials against RHE and Hg/HgO, respectively.

The electrochemical double-layer capacitance (C_{dl}) was used to estimate the electrochemical surface area (ECSA) of the electrocatalyst. C_{dl} was obtained by CV method at various scanning rates (1, 3, 5, 7, 9, and 10 mV s^{-1}) in 1.0 M KOH containing 0.33 M urea. In-situ EIS tests were conducted with a three-electrode system within a a frequency range of 10^{-1} to 10^5 Hz with an AC amplitude of 10 mV. The measured potentials are 1.10, 1.15, 1.20, 1.25, 1.30, 1.35 and 1.40 V for UOR. For HER, the measured potentials are 0.2, 0.1, 0, -0.1, -0.2, -0.25, -0.3, -0.35, -0.4 and -0.45 V.

The TOF value was calculated by the equation:

$$TOF = \frac{j \times A}{n \times F \times N} \qquad \text{(S2)}$$

where j is the current density. A is the geometric area, n represents the electrons transfer numbers in the process of UOR and HER. F is the Faradaic constant (96485 C mol−1). and N is the number of active sites. The N value was determined using CV. CV measurements were performed in 1.0 M KOH solution at a scan rate of 50 mV s^{-1}, within the potential range of 0-0.6 V vs RHE. The integrated charge of each CV curve was calculated over the entire potential range, and the half value of this integrated charge was defined as Q. The N value was then calculated using the equation: N = Q/2F.

To measure the performances of the two-electrode electrolysis, the as-prepared catalysts were used as both anode and cathode. LSV curves were recorded at a scan rate of 5 mV s^{-1} in 1.0 M KOH with and without 0.33 M urea addition. For the anion exchange membrane (AEM) flow electrolyzer, a Fumasep FAA-3-PK-130 anionic membrane was purchased from Suzhou Shengernuo Co., LTD. The membrane was activated by soaking it in 1.0 M KOH solution for 24 h prior to use. The membrane was sandwiched between the anode and cathode (effective electrode area: 1.0 × 1.0 cm^2). 1.0 M KOH with and without 0.33 M urea/artificial urine electrolytes were fed into the AEM flow electrolyzer through pump (Flow rate: 10 mL min^{-1}) at room temperature (25℃). Additionally, the 60 ℃ test was conducted in a thermostatic chamber. To minimize errors and uncertainties, the electrochemical performance test results were repeatedly verified.

4. Faradaic efficiency measurement

Chronopotentiometry was performed at a current density of 1 A cm^{-2} to continuously produce H_2 and anode gaseous products. The generated H_2 was collected by the drainage method and recorded every 5 minutes. The anode gaseous products during UOR at 1000 mA cm^{-2} were analyzed using a gas chromatograph (GC9790 Plus, Fuli Analytical Instrument Co., Ltd.) equipped with a MolSieve 5A column and a thermal conductive detector (TCD), employing argon as the carrier gas. The injection volume for the analysis was 0.1 mL.

The Faradaic efficiency (FE) of H_2 and anode gaseous products was calculated

according to the following formula:

$$FE = \frac{n_{measured}}{n_{theroetical}} = \frac{PV/RT}{Q/zF} \tag{S3}$$

Where P is the pressure (1 atm), V is the volume of H_2 generated, R is the gas constant (8.3144621 J mol^{-1} K^{-1}), T is the temperature, Q is the charge, z is the number of stoichiometric charges (the values of z for H_2, O_2 and N_2 are 2, 4 and 6), and F is the Faraday constant (96485.34 C mol^{-1}).

Ion chromatography (IC) was used to determine the concentration of nitrate and nitrite. The instrument was Qingdao Shenghan CIC-D160+, eluent generator was used, KOH was used as eluent (isocratic elution: the concentration was 15 mM), and the pump flow rate was 0.70 mL min^{-1}, and the column temperature was 35 °C. The single standard external standard method was used for secondary fitting. The liquid sample was diluted and passed through the column, and the sample was injected 25 μL at a time.

The quantitative analysis of nitrate and nitrite was performed using an IC detector. The FE of the liquid products was calculated according to the following formula[1]:

$$FE = \frac{z \times V \times c \times F}{Q \times M_w} \tag{S4}$$

where z is a number of the electrons transferred (z = 6 for NO_2^- and 8 for NO_3^-), F is the Faraday constant, c is the concentration of nitrate/nitrate in the analyte in ppm, V is the total volume of the anolyte, Q is the total charge passed, and Mw is the molecular weight of nitrate/nitrate.

5. Density functional theory calculations

DFT calculations were applied based on the Vienna Ab-initio Simulation Package (VASP). The generalized gradient approximation (GGA) and Perdew-Burke-Ernzerhof (PBE) was selected for the electronic exchange-correlation energy. The convergence criterion for geometry optimizations was set to not exceed 0.0015 eV·$Å^{-1}$ in force on each atom. The energy cutoff for plane wave-basis was set to 400 eV to describe the orbitals. The pair-wise interatomic interaction strength was analyzed using the Crystal

orbital Hamilton population (COHP) with the Lobster 4.1.0 code and the basis set of Koga. The Monkhorst-Pack scheme with a (2 × 2 ×1) mesh were employed for k-points sampling. A vacuum layer of at least 15 Å was included in the calculations.

6. Supplementary Results

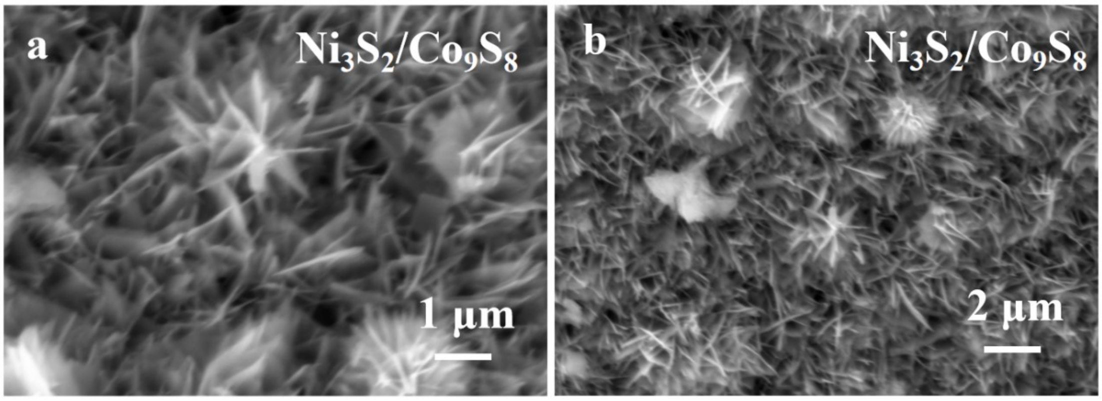

Figure S1. a, b) SEM images of Ni_3S_2/Co_9S_8.

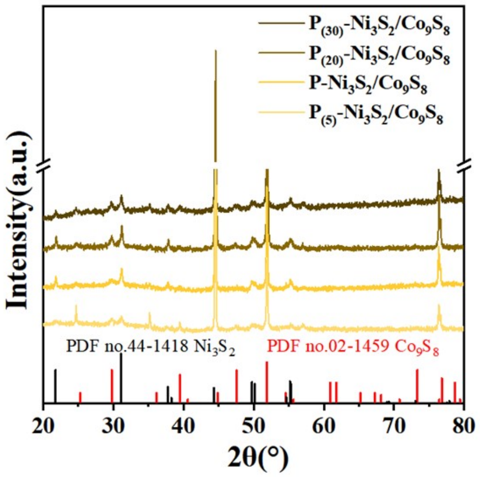

Figure S2. XRD patterns of the Ni_3S_2/Co_9S_8 with different content of P ($P_{(5)}$-Ni_3S_2/Co_9S_8, P-Ni_3S_2/Co_9S_8, $P_{(20)}$-Ni_3S_2/Co_9S_8 and $P_{(30)}$-Ni_3S_2/Co_9S_8).

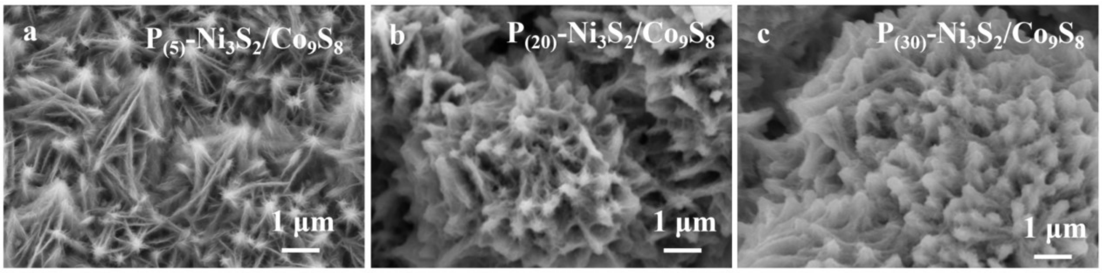

Figure S3. SEM images of a) $P_{(5)}$-Ni_3S_2/Co_9S_8, b) $P_{(20)}$-Ni_3S_2/Co_9S_8 c) $P_{(30)}$-Ni_3S_2/Co_9S_8.

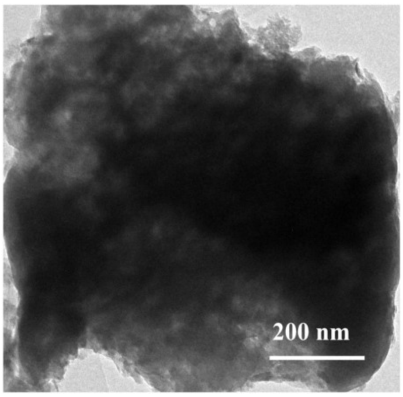

Figure S4. TEM image of P-Ni_3S_2/Co_9S_8.

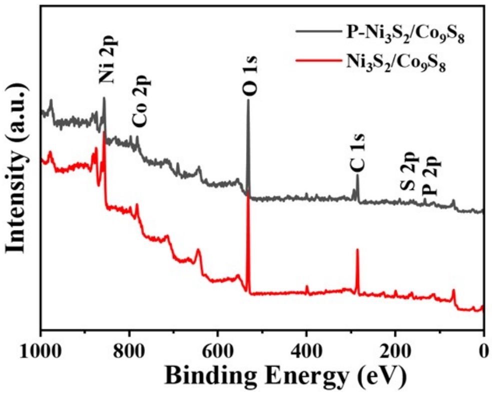

Figure S5. XPS survey spectra for Ni_3S_2/Co_9S_8 and P-Ni_3S_2/Co_9S_8.

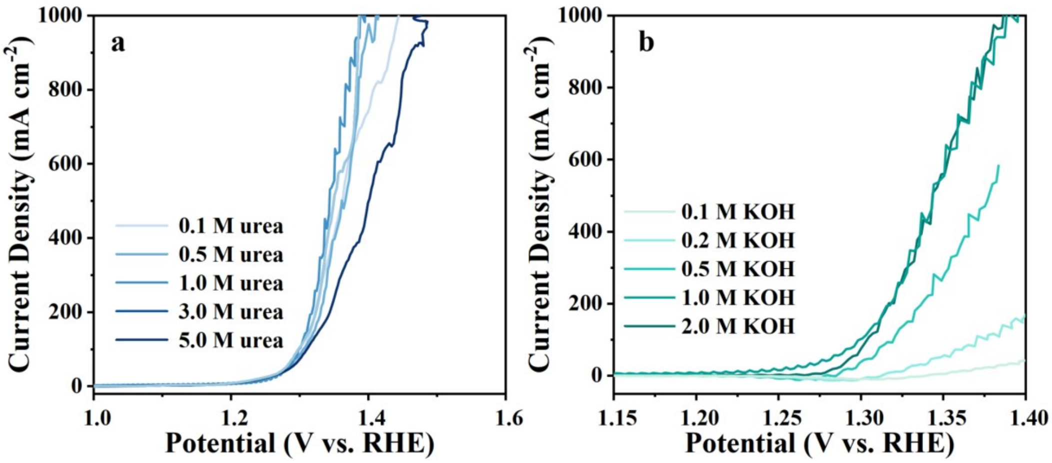

Figure S6. LSV curves of P-Ni_3S_2/Co_9S_8 in a) different concentrations of urea with 1.0 M KOH and b) different concentrations of KOH with 0.33 M urea.

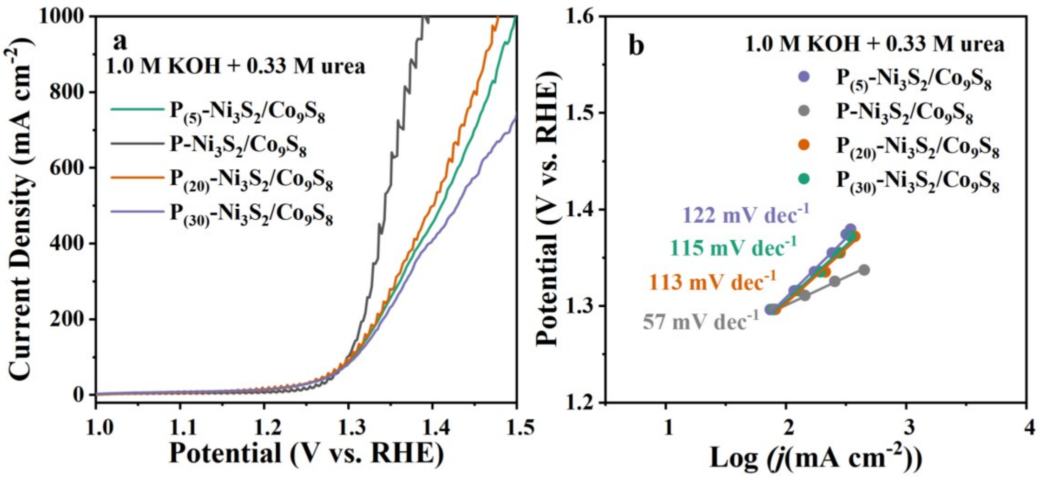

Figure S7. a) LSV curves of P-Ni_3S_2/Co_9S_8 with different doping amounts of P in 1.0 M KOH with 0.33 M urea and the corresponding b) Tafel slopes.

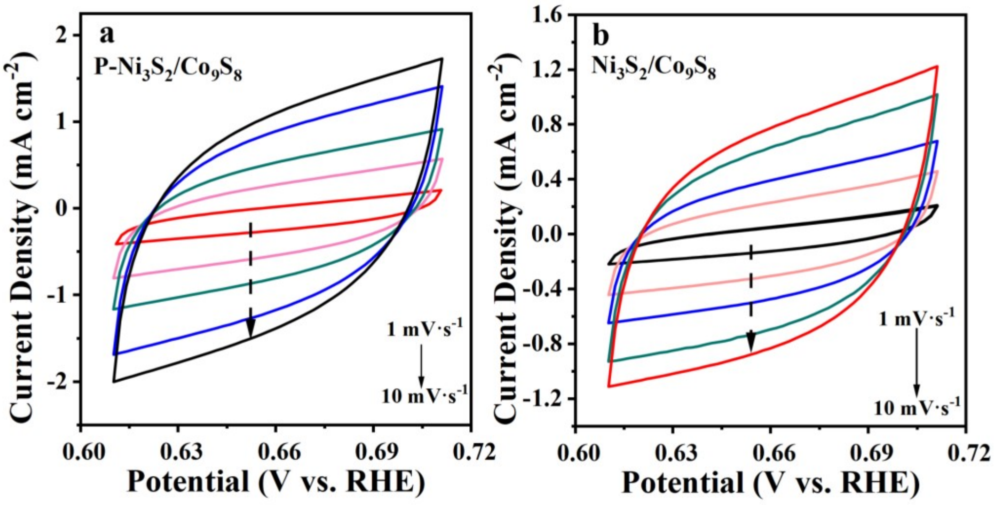

Figure S8. CV curves of a) P-Ni_3S_2/Co_9S_8, b) Ni_3S_2/Co_9S_8 for UOR at different scan rates.

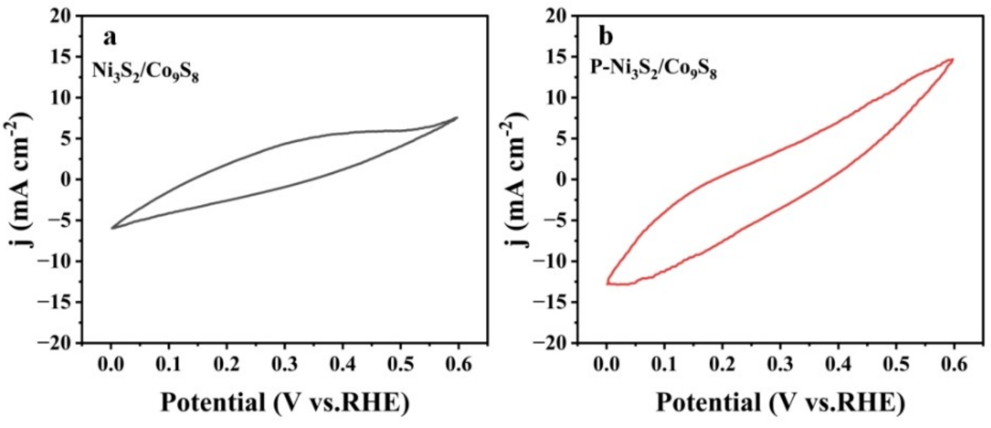

Figure S9. CV curves of a) P-Ni_3S_2/Co_9S_8, b) Ni_3S_2/Co_9S_8.

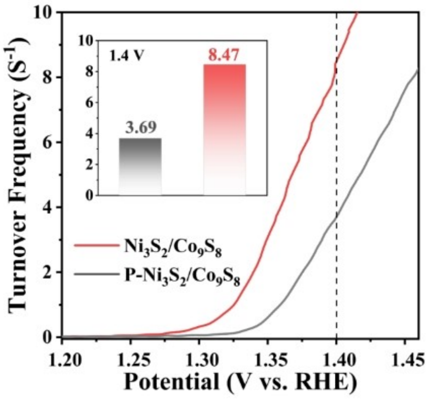

Figure S10.The TOF values of the P-Ni_3S_2/Co_9S_8, Ni_3S_2/Co_9S_8 for UOR.

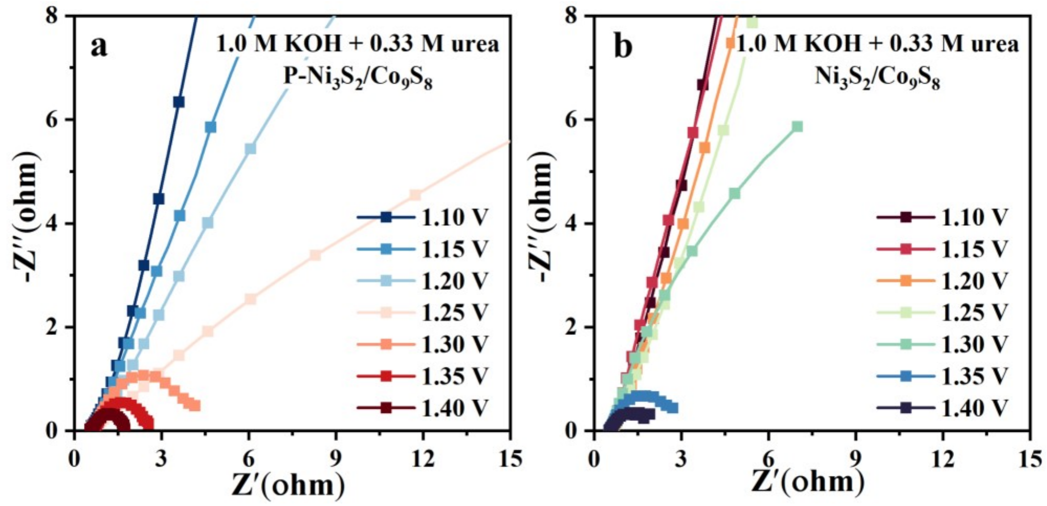

Figure S11. The Nyquist plots of a) P-Ni_3S_2/Co_9S_8 and b) Ni_3S_2/Co_9S_8 at various voltages in 1.0 M KOH and 0.33 M Urea.

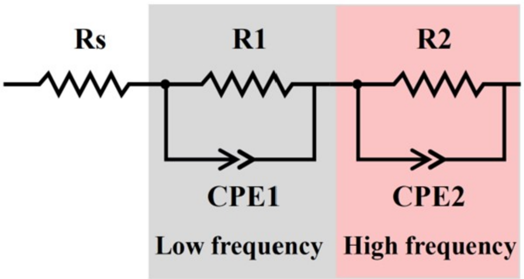

Figure S12. The equivalent circuit model of P-Ni_3S_2/Co_9S_8 and Ni_3S_2/Co_9S_8.

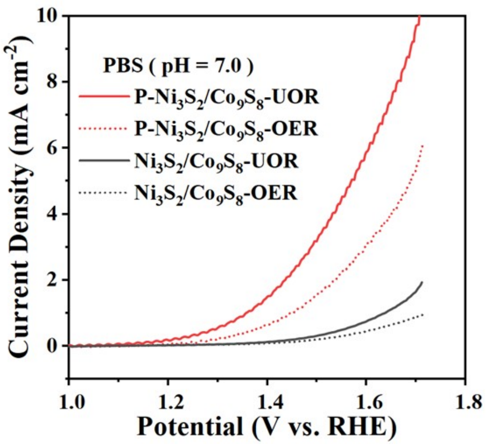

Figure S13. The LSV curves of P-Ni_3S_2/Co_9S_8 and Ni_3S_2/Co_9S_8 in phosphate buffered saline (PBS, pH = 7.0).

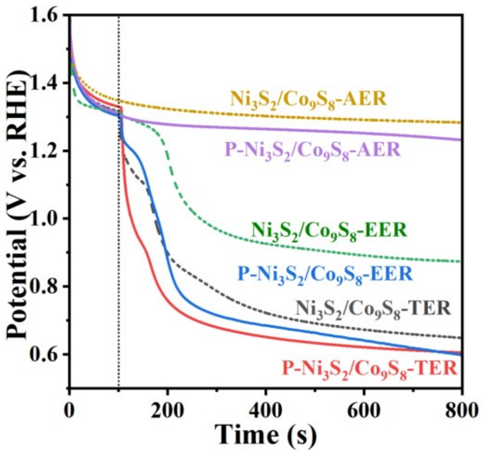

Figure S14. The OCP of P-Ni_3S_2/Co_9S_8 and Ni_3S_2/Co_9S_8 in 1.0 M KOH solution before and after the injection of Acetamide, Thiourea and Ethylenediamine.

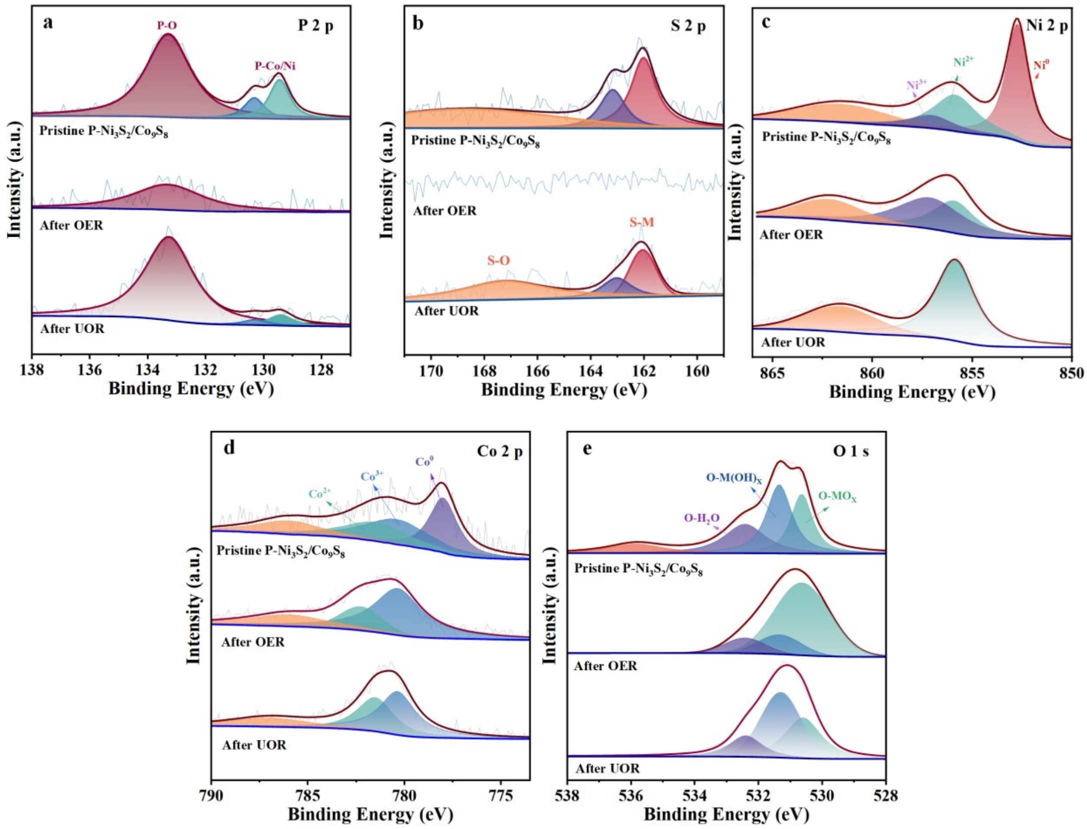

Figure S15. XPS spectra of a) P 2p, b) S 2p, c) Ni 2p, d) Co 2p and e) O 1s for P-Ni_3S_2/Co_9S_8, P-Ni_3S_2/Co_9S_8 after OER and after UOR.

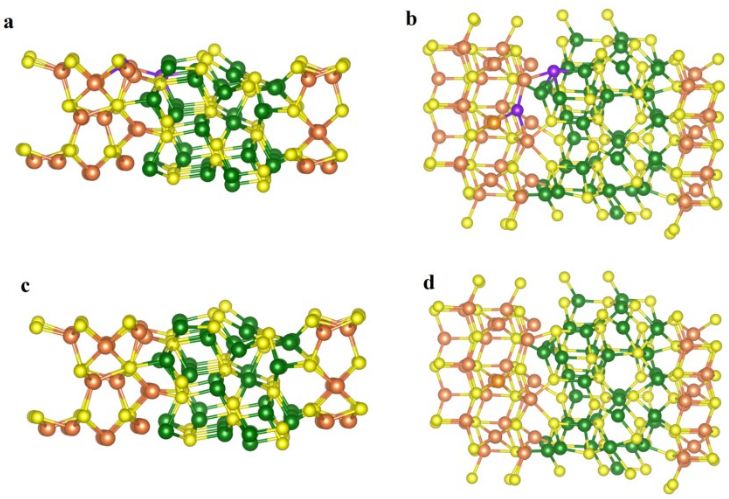

Figure S16. Three optimized adsorption structures of a, b) Ni_3S_2/Co_9S_8 and c, d) P-Ni_3S_2/Co_9S_8. The Gray, blue, red, white, purple, yellow, orange and green balls represent C, N, O, H, P, S, Ni and Co atoms, respectively.

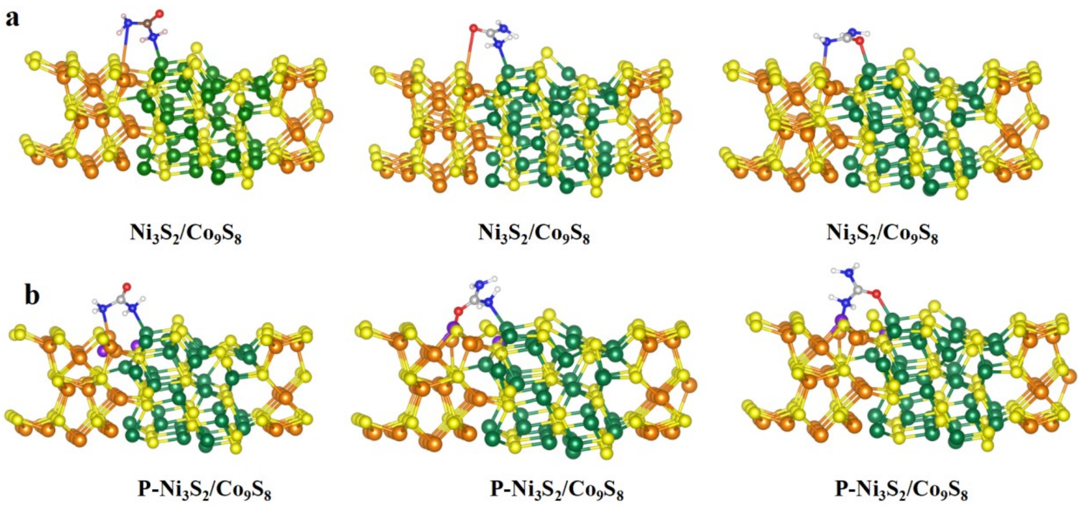

Figure S17. Three optimized adsorption structures for $CO(NH_2)_2$ on a) Ni_3S_2/Co_9S_8 and b) P-Ni_3S_2/Co_9S_8. The gray, blue, red, white, purple, yellow, orange, and green balls represent C, N, O, H, P, S, Ni and Co atoms, respectively.

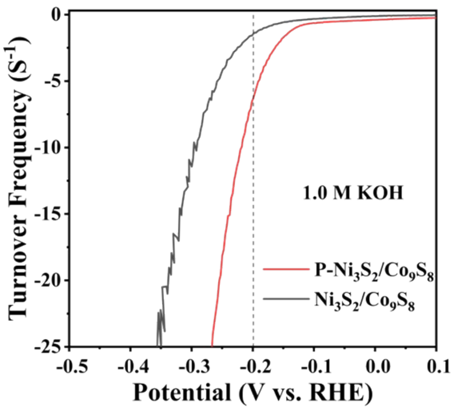

Figure S18. TOF values of the P-Ni_3S_2/Co_9S_8, Ni_3S_2/Co_9S_8 for HER.

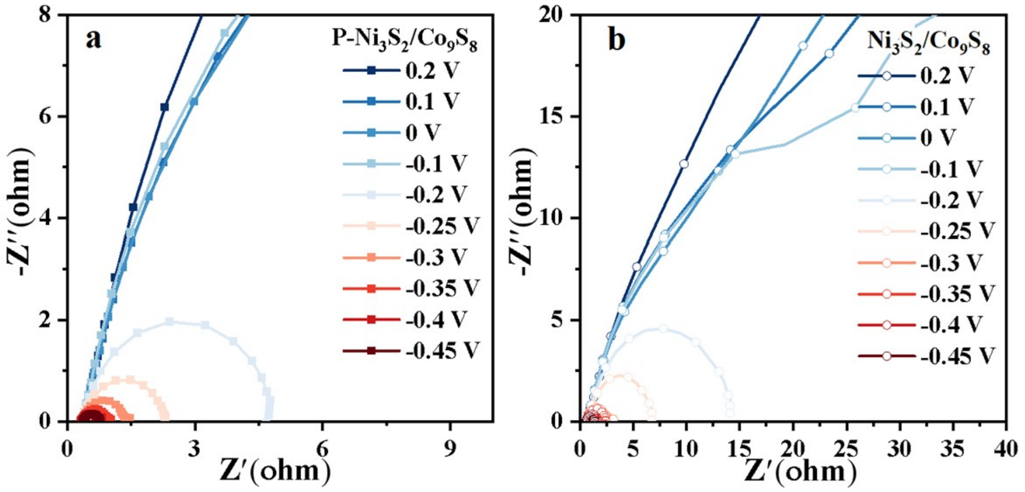

Figure S19. In-situ EIS characterization of the Nyquist plots of a) P-Ni_3S_2/Co_9S_8 and b) Ni_3S_2/Co_9S_8 at various voltages during HER.

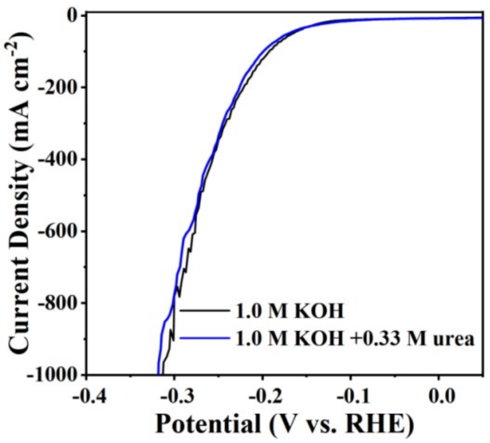

Figure S20. LSV curves of P-Ni_3S_2/Co_9S_8 during HER at 1.0 M KOH/1.0 M KOH contain 0.33 M urea.

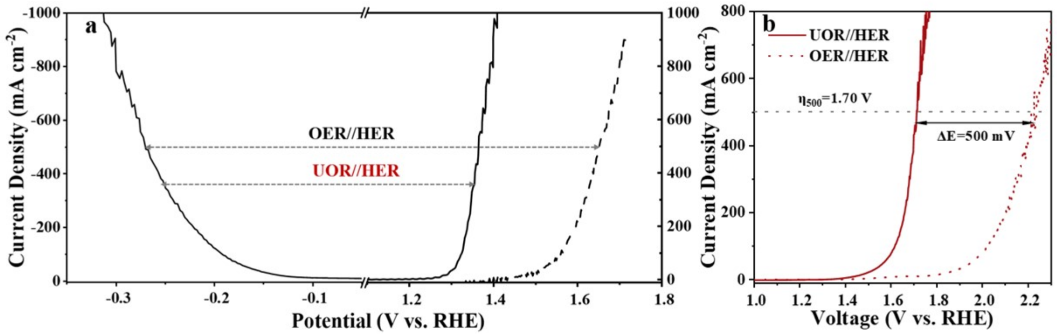

Figure S21. LSV curves of P-Ni_3S_2/Co_9S_8//P-Ni_3S_2/Co_9S_8 during a) UOR//HER and b)OER//HER.

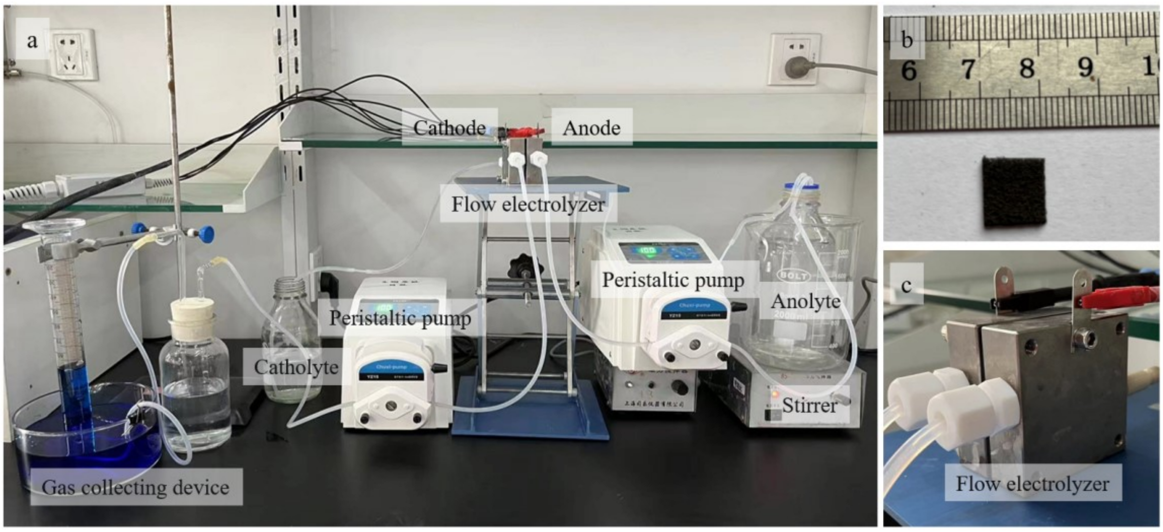

Figure S22. a) Electrolysis setup of the UOR//HER in anion exchange membrane flow electrolyzer. b) P-Ni_3S_2/Co_9S_8 electrode. c) The anion exchange membrane flow electrolyzer (Flow rate: 10 mL min^{-1}).

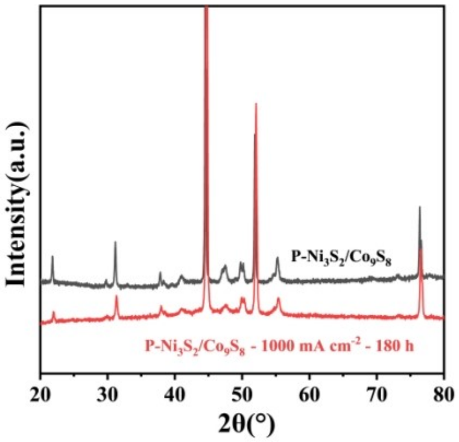

Figure S23. XRD patterns of P-Ni_3S_2/Co_9S_8 before and after durability UOR test at 1000 mA cm^{-2} for 180 h

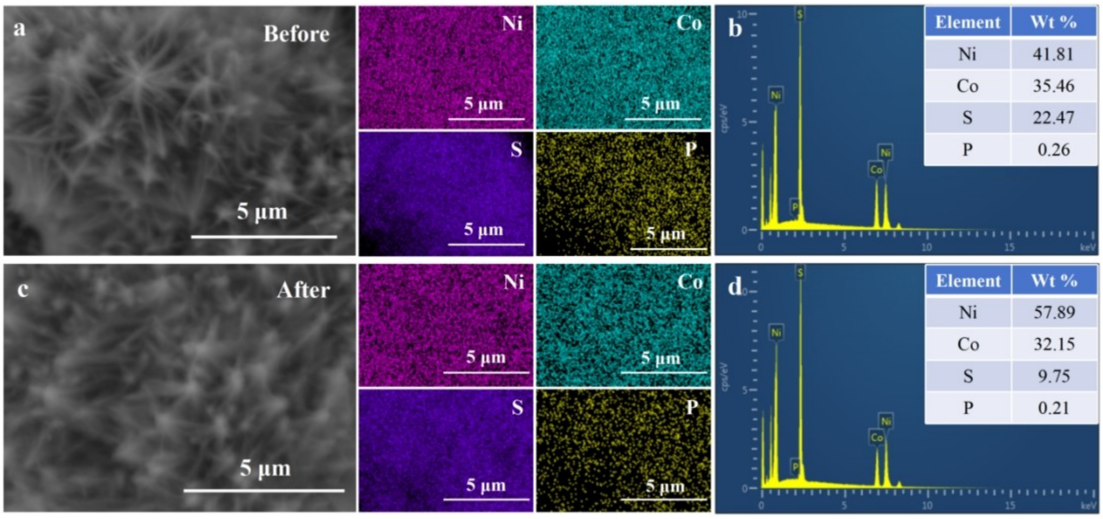

Figure S24. SEM images and corresponding elemental mapping images and EDS data of P-Ni_3S_2/Co_9S_8 a, b) before and c, d) after the durability test for UOR at 1000 mA cm^{-2} for 180 h

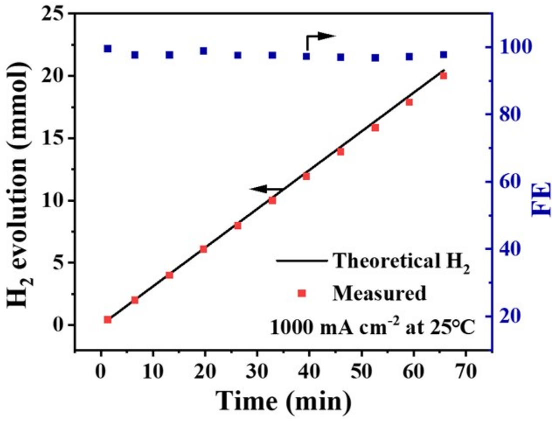

Figure S25. The theoretical and measured H_2 evolution amount and FE at a constant current of 1000 mA cm^{-2} at a) 25 °C

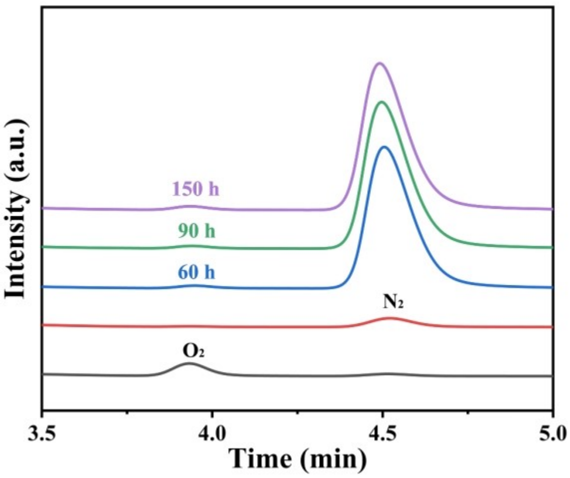

Figure S26. GC trace of the gaseous products at different time compared with several standard gases

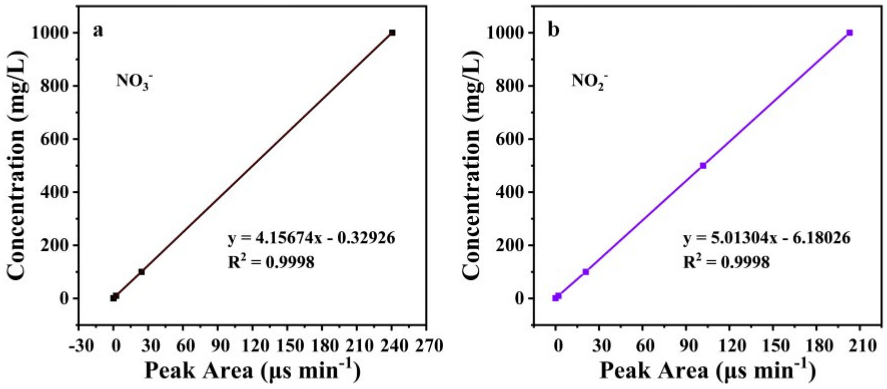

Figure S27. Calibration curve for a) NO_3^- and b) NO_2^-

Table S1. Comparison of UOR and HER performance for P-Ni_3S_2/Co_9S_8 and recently reported electrocatalysts.

Catalysts	UOR performances	C_{Urea}	HER performances	References
**P-Ni_3S_2/Co_9S_8**	**1.22 V@10 mA cm^{-2} 1.30 V@ 100 mA cm^{-2}**	**0.33M**	**66 V@10 mA cm^{-2} 194V@100 mA cm^{-2}**	**This work**
NF/NiMoO-Ar	1.37 V@10 mA cm^{-2} 1.42 V@100 mA cm^{-2}	0.5M	-	[2]

$Ni_2P_4O_{12}$/NiTe	1.313 V@ 10 mA cm^{-2}	0.33 M	24 V@10 mA cm^{-2}	[3]
NCP/CC	1.37 V@ 100 mA cm^{-2}	0.33M		[4]
Ni_3S_2-Ni_3P/NF	1.38 V@ 100 mA cm^{-2}	0.5M	122 V@10 mA cm^{-2}<br>256 V@100 mA cm^{-2}	[5]
NiCoCr-LDH/NF	1.33 V@ 10 mA cm^{-2}<br>1.38 V@100 mA cm^{-2}	0.5M	-	[6]
Ni_2P/Fe_2P	1.36 V@10 mA cm^{-2}	0.5M	115 V@10 mA cm^{-2}	[7]
Ni-Mo alloy	1.36 V@10 mA cm^{-2}	0.1M	44 V@10 mA cm^{-2}	[8]
FQD/CoNi-LDH/NF	1.46 V@10 mA cm^{-2}<br>1.42 V@100 mA cm^{-2}	0.5M	70 V@10 mA cm^{-2}<br>230 V@100 mA cm^{-2}<br>330 V@200 mA cm^{-2}	[9]
Ni-Co_9S_8	1.28 V@10 mA cm^{-2}<br>1.43 V@100 mA cm^{-2}	0.33M	295 V@100 mA cm^{-2}	[10]
NiS@Ni_3S_2/$NiMoO_4$	1.3 V@10 mA cm^{-2}	0.5M	80 V@10 mA cm^{-2}	[11]
P-$CoNi_2S_4$	1.306 V@10 mA cm^{-2}<br>1.367 V@100 mA cm^{-2}	0.5M	135 V@10 mA cm^{-2}	[12]
Ni_3S_2/NiS	1.273 V@10 mA cm^{-2}	0.5M	146 V@10 mA cm^{-2}	[13]
$Ni_3N/Ni_{0.2}Mo_{0.8}N$	1.328 V@10 mA cm^{-2}<br>1.366 V@100 mA cm^{-2}<br>1.406 V@200 mA cm^{-2}	0.5M	55 V@10 mA cm^{-2}	[14]
NiFeOOH/NF	1.38 V@50 mA cm^{-2}<br>1.41 V@100 mA cm^{-2}	0.33M	-	[15]
Ni/NiO-N-C-500	1.38 V@10 mA cm^{-2}	0.33 M	-	[16]
$Co_2Mo_3O_8$	1.40 V@100 mA cm^{-2}	0.5M	37 V@10 mA cm^{-2}<br>140 V@100 mA cm^{-2}	[17]
Ni-S-Se	1.38 V@10 mA cm^{-2}<br>1.42 V@100 mA cm^{-2}	0.5 M	98 V@10 mA cm^{-2}<br>206 V@100 mA cm^{-2}	[18]
O-NiMoP/NF	1.41 V@100 mA cm^{-2}	0.5 M	54 V@10 mA cm^{-2}	[19]
V-Ni_3N/NF	1.361 V@10 mA cm^{-2}	0.5 M	83 V@10 mA cm^{-2}	[20]
Ni_2P/NiMoP	1.33 V@10 mA cm^{-2}	0.33 M	22 V@10 mA cm^{-2}<br>91 V@100 mA cm^{-2}	[21]
$NiMoO_4$-300/NF	1.36 V@10 mA cm^{-2}	0.5 M	68 V@10 mA cm^{-2}	[22]
NiS nanotubes	1.39 V@100 mA cm^{-2}	0.33 M	-	[23]
Mo-NiS	1.33 V@10 mA cm^{-2}<br>1.355 V@100 mA cm^{-2}	0.5 M	174 V@10 mA cm^{-2}	[24]
Co_3O_4/Ti_3C_2	1.40 V@10 mA cm^{-2}	0.5M	124 V@10 mA cm^{-2}	[25]
CoP@PNC/PCWF	1.29 V@10 mA cm^{-2}<br>1.32 V@50 mA cm^{-2}	0.5M	-	[26]
Fe-($Ni_{12}P_5/Ni_3P$)	1.304 V@10 mA cm^{-2}<br>1.419 V@100 mA cm^{-2}	0.5 M	-	[27]

NiCo-BDC/Ni-S	1.31 V@10 mA cm^{-2}	0.33M	-	[28]
N-Co_9S_8/Ni_3S_2/NF	1.35 V@50 mA cm^{-2}<br>1.41 V@100 mA cm^{-2}<br>1.47 V@400 mA cm^{-2}	0.5M	111 V@10 mA cm^{-2}	[29]
Superthin amorphous nickel hydroxide (ANH)	1.34 V@100 mA cm^{-2}	0.33M	-	[30]

Table S2. Comparison of P-Ni_3S_2/Co_9S_8 properties with previously reported catalysts in flow electrolyzers for urea or urine oxidation coupled with HER

**Catalysts**	**Operation time (h)@Current density (mA cm^{-2})**	**Temperature (°C)**	**Voltage retention (%)**	**Electrolyte**	**Decay rate (%/h)**	**Ref.**
**P-Ni_3S_2/Co_9S_8**	**180@1000**<br>**180@1000**	**25**<br>**60**	**92.1%**<br>**93.5%**	**0.33 M Urine**	**0.044%/h**<br>**0.036%/h**	**This work**
NiCoCr-LDH/NF	50@~23	25	95.3%	0.5 M Urea	0.094%/h	[31]
$CoS_{1.097}$/Ni_3S_2	60@100	25	95%	0.33 M Urea	0.083%/h	[32]
Co-FeOOH	24@1000	25	87.6%	0.33 M Urea	0.517%/h	[33]
Mo-NiS	12@50	25	95.8	0.5 M Urea	0.087%/h	[34]

Table S3. ICP-AES analysis of the total Ni and Co in the electrolyte and P-Ni_3S_2/Co_9S_8 after the durability test for UOR at 1000 mA cm^{-2} for 180 h

**Element content[a]**	**Ni (ug cm^{-2})**	**Co (ug cm^{-2})**
P-Ni_3S_2/Co_9S_8	389.6 ± 5.652	125.6 ± 5.652
Electrolyte	0.027 ± 0.016	0.625 ± 0.023

a.element content is normalized based on the geometric area of the electrode.

Table S4. The FEs of O_2 and N_2 in the anode products during the stability (1000 mA cm^{-2}) test at different time intervals

**Time**	**FE for O_2 (%)**	**FE for N_2 (%)**
60 h	0.48	74.63
90 h	0.55	69.76
150 h	0.51	69.47

References

1. S. W. Tatarchuk, J. J. Medvedev, F. Li, Y. Tobolovskaya, A. Klinkova, Nickel-Catalyzed Urea Electrolysis: From Nitrite and Cyanate as Major Products to Nitrogen Evolution. Angewandte Chemie International Edition, 2022, **61**, e202209839.

2. Z. Y. Yu, C. C. Lang, M. R. Gao, Y. Chen, Q. Q. Fu, Y. Duan and S. H. Yu, Ni–Mo–O nanorod-derived composite catalysts for efficient alkaline water-to-hydrogen conversion via urea electrolysis, *Energy Environ. Sci.*, 2018, **11**, 1890-1897.

3. P. Guo, S. Cao, W. Huang, X. Lu, W. Chen, Y. Zhang, Y. Wang, X. Xin, R. Zou, S. Liu and X. Li, Heterojunction-Induced Rapid Transformation of Ni^{3+}/Ni^{2+} Sites which Mediates Urea Oxidation for Energy-Efficient Hydrogen Production, *Adv. Mater.*, 2024, **36**, 2311766.

4. H.-J. Zhang, Z.-Q. Chen, X.-T. Ye, K. Xiao and Z.-Q. Liu, Electron Delocalized Ni Active Sites in Spinel Catalysts Enable Efficient Urea Oxidation, *Angew. Chem. Int. Ed.*, 2024, **64**, e202421027.

5. J. Liu, Y. Wang, Y. Liao, C. Wu, Y. Yan, H. Xie and Y. Chen, Heterostructured Ni_3S_2-Ni_3P/NF as a Bifunctional Catalyst for Overall Urea-Water Electrolysis for Hydrogen Generation, *ACS Appl. Mater. Interfaces*, 2021, **13**, 26948-26959.

6. S. Xu, D. Jiao, X. Ruan, Z. Jin, Y. Qiu, Z. Feng, L. Zheng, J. Fan, W. Zheng and X. Cui, O-2p Hybridization Enhanced Transformation of Active γ-NiOOH by Chromium Doping for Efficient Urea

Oxidation Reaction, *Adv. Funct. Mater.*, 2024, DOI: 10.1002/adfm.202401265, 2401265.

7. L. Yan, Y. Sun, E. Hu, J. Ning, Y. Zhong, Z. Zhang and Y. Hu, Facile in-situ growth of Ni_2P/Fe_2P nanohybrids on Ni foam for highly efficient urea electrolysis, *J. Colloid Interface Sci.*, 2019, **541**, 279-286.

8. J. Y. Zhang, T. He, M. Wang, R. Qi, Y. Yan, Z. Dong, H. Liu, H. Wang and B. Y. Xia, Energy-saving hydrogen production coupling urea oxidation over a bifunctional nickel-molybdenum nanotube array, *Nano Energy*, 2019, **60**, 894-902.

9. Y. Feng, X. Wang, J. Huang, P. Dong, J. Ji, J. Li, L. Cao, L. Feng, P. Jin and C. Wang, Decorating CoNi layered double hydroxides nanosheet arrays with fullerene quantum dot anchored on Ni foam for efficient electrocatalytic water splitting and urea electrolysis, *Chem. Eng. J.*, 2020, **390**, 124525.

10. P. Hao, W. Zhu, L. Li, J. Tian, J. Xie, F. Lei, G. Cui, Y. Zhang and B. Tang, Nickel incorporated Co_9S_8 nanosheet arrays on carbon cloth boosting overall urea electrolysis, *Electrochim. Acta*, 2020, **338**, 135883.

11. L. Sha, T. Liu, K. Ye, K. Zhu, J. Yan, J. Yin, G. Wang and D. Cao, A heterogeneous interface on $NiS@Ni_3S_2/NiMoO_4$ heterostructures for efficient urea electrolysis, *J. Mater. Chem. A*, 2020, **8**, 18055-18063.

12. X. F. Lu, S. L. Zhang, W. L. Sim, S. Gao and X. W. D. Lou, Phosphorized $CoNi_2S_4$ Yolk-Shell Spheres for Highly Efficient Hydrogen Production via Water and Urea Electrolysis, *Angew. Chem. Int. Ed.*, 2021, **60**, 22885-22891.

13. Q. Zhao, C. Meng, D. Kong, Y. Wang, H. Hu, X. Chen, Y. Han, X. Chen, Y. Zhou, M. Lin and M. Wu, In Situ Construction of Nickel Sulfide Nano-Heterostructures for Highly Efficient Overall Urea Electrolysis, *ACS Sustainable Chem. Eng.*, 2021, **9**, 15582-15590.

14. R. Q. Li, X. Y. Wan, B. L. Chen, R. Y. Cao, Q. H. Ji, J. Deng, K. G. Qu, X. B. Wang and Y. C. Zhu, Hierarchical $Ni_3N/Ni_{0.2}Mo_{0.8}N$ heterostructure nanorods arrays as efficient electrocatalysts for overall water and urea electrolysis, *Chem. Eng. J.*, 2021, **409**, 128240.

15. P. Babar, K. Patil, D. M. Lee, V. Karade, K. Gour, S. Pawar and J. H. Kim, Cost-effective and efficient water and urea oxidation catalysis using nickel-iron oxyhydroxide nanosheets synthesized by an ultrafast method, *J. Colloid Interface Sci.*, 2021, **584**, 760-769.

16. N. Wu, R. Guo, X. Zhang, N. Gao, X. Chi, D. Cao and T. Hu, Nickel/nickel oxide nanocrystal nitrogen-doped carbon composites as efficient electrocatalysts for urea oxidation, *J. Alloys Compd.*,

2021, **870**, 159408.

17. K. Zhang, C. Liu, N. Graham, G. Zhang and W. Yu, Modulation of dual centers on cobalt-molybdenum oxides featuring synergistic effect of intermediate activation and radical mediator for electrocatalytic urea splitting, *Nano Energy*, 2021, **87**, 106217.

18. N. Chen, Y.-X. Du, G. Zhang, W.-T. Lu and F.-F. Cao, Amorphous nickel sulfoselenide for efficient electrochemical urea-assisted hydrogen production in alkaline media, *Nano Energy*, 2021, **81**, 105605.

19. H. Jiang, M. Sun, S. Wu, B. Huang, C. S. Lee and W. Zhang, Oxygen-Incorporated NiMoP Nanotube Arrays as Efficient Bifunctional Electrocatalysts For Urea-Assisted Energy-Saving Hydrogen Production in Alkaline Electrolyte, *Adv. Funct. Mater.*, 2021, **31**, 2104951.

20. R. Q. Li, Q. Liu, Y. Zhou, M. Lu, J. Hou, K. Qu, Y. Zhu and O. Fontaine, 3D self-supported porous vanadium-doped nickel nitride nanosheet arrays as efficient bifunctional electrocatalysts for urea electrolysis, *J. Mater. Chem. A*, 2021, **9**, 4159-4166.

21. T. Wang, X. Cao and L. Jiao, Ni_2P/NiMoP heterostructure as a bifunctional electrocatalyst for energy-saving hydrogen production, *eScience*, 2021, **1**, 69-74.

22. C. Chen, S. He, K. Dastafkan, Z. Zou, Q. Wang and C. Zhao, Sea urchin-like $NiMoO_4$ nanorod arrays as highly efficient bifunctional catalysts for electrocatalytic/photovoltage-driven urea electrolysis, *Chin. J. Catal.*, 2022, **43**, 1267-1276.

23. M. Zhong, W. Li, C. Wang and X. Lu, Synthesis of hierarchical nickel sulfide nanotubes for highly efficient electrocatalytic urea oxidation, *Appl. Surf. Sci.*, 2022, **575**, 151708.

24. Y. Zhou, Y. Wang, D. Kong, Q. Zhao, L. Zhao, J. Zhang, X. Chen, Y. Li, Y. Xu and C. Meng, Revealing the Reactant Mediation Role of Low-Valence Mo for Accelerated Urea-Assisted Water Splitting, *Adv. Funct. Mater.*, 2023, **33**, 2210656.

25. Y. Zhang, Z. Zhang, A. Addad, Q. Wang, P. Roussel, M. A. Amin, S. Szunerits and R. Boukherroub, 0D/2D Co_3O_4/Ti_3C_2 MXene Composite: A Dual-Functional Electrocatalyst for Energy-Saving Hydrogen Production and Urea Oxidation, *ACS Appl. Energy Mater.*, 2022, **5**, 15471-15482.

26. J. Kang, F. Yang, C. Sheng, H. Xu, J. Wang, Y. Qing, Y. Wu and X. Lu, CoP Nanoparticle Confined in P, N Co-Doped Porous Carbon Anchored on P-Doped Carbonized Wood Fibers with Tailored Electronic Structure for Efficient Urea Electro-Oxidation, *Small*, 2022, **18**, 2200950.

27. X. Xu, C. Zhang, J. Li, H. Liu, G. Su, Z. Shi and M. Huang, Redistributing interfacial charge density of $N_{i12}P_5/Ni_3P$ via Fe doping for ultrafast urea oxidation catalysis at large current densities, *Chem. Eng.*

J., 2023, **452**, 139362.

28. Y. Chen, X. Zeng, Q. Meyer, C. Zhao, Z. He, F. Wu, H. Tang and Y. Cheng, An outstanding NiFe/NF oxygen evolution reaction boosted by the hydroxyl oxides, *FlatChem*, 2023, **442**, 100222.

29. H. Xie, Y. Feng, X. He, Y. Zhu, Z. Li, H. Liu, S. Zeng, Q. Qian and G. Zhang, Construction of Nitrogen-Doped Biphasic Transition-Metal Sulfide Nanosheet Electrode for Energy-Efficient Hydrogen Production via Urea Electrolysis, *Small*, 2023, **19**, 2207425.

30. Y. Zhu, C. Liu, S. Cui, Z. Lu, J. Ye, Y. Wen, W. Shi, X. Huang, L. Xue, J. Bian, Y. Li, Y. Xu and B. Zhang, Multistep Dissolution of Lamellar Crystals Generates Superthin Amorphous $Ni(OH)_2$ Catalyst for UOR, *Adv. Mater.*, 2023, **35**, 2301549.

31. S. Xu, D. Jiao, X. Ruan, Z. Jin, Y. Qiu, Z. Feng, L. Zheng, J. Fan, W. Zheng and X. Cui, O-2p Hybridization Enhanced Transformation of Active γ-NiOOH by Chromium Doping for Efficient Urea Oxidation Reaction, *Adv. Funct. Mater.*, 2024, **36**, 2401265.

32. M. Du, Y. Ji, Y. Li, S. Liu and J. Yan, Construction of an Internal Charge Field: $CoS_{1.097}/Ni_3S_2$ Heterojunction Promotes Efficient Urea Oxidation Reaction, *Adv. Funct. Mater.*, 2024, **38**, 2402776.

33. G. Liang, R. Zhang, C. Ji, C. Wang, L. Zhang, X. Long, C. Li, D. Li and D. Yang, Electron Shuttling of Iron-Oxygen-Cobalt Bridging in Cobalt Assembled Iron Oxyhydroxide Catalyst Boosts the Urea Oxidation Stability and Activity, *Adv. Funct. Mater.*, 2025. DOI: 10.1002/adfm.202501170.

34. Y. Zhou, Y. Wang, D. Kong, Q. Zhao, L. Zhao, J. Zhang, X. Chen, Y. Li, Y. Xu, and C. Meng, "Revealing the reactant mediation role of low-valence Mo for accelerated urea-assisted water splitting," Adv. Funct. Mater., 2022, **33**, 2210656.
